# Non-invasive Brain Stimulation for the Treatment of Gilles de la Tourette Syndrome

**DOI:** 10.3389/fneur.2020.592258

**Published:** 2020-11-10

**Authors:** Maximilian Kleimaker, Alexander Kleimaker, Anne Weissbach, Lorenza S. Colzato, Christian Beste, Tobias Bäumer, Alexander Münchau

**Affiliations:** ^1^Institute of Systems Motor Science, University of Lübeck, Lübeck, Germany; ^2^Department of Neurology, University Hospital Schleswig-Holstein, Lübeck, Germany; ^3^Cognitive Neurophysiology, Department of Child and Adolescent Psychiatry, Faculty of Medicine, Technische Universität Dresden, Dresden, Germany

**Keywords:** Gilles de la Tourette syndrome, transcranial magnetic stimulation, transcranial direct current stimulation, state informed brain stimulation, brodmann area 40

## Abstract

Gilles de la Tourette Syndrome is a multifaceted neuropsychiatric disorder typically commencing in childhood and characterized by motor and phonic tics. Its pathophysiology is still incompletely understood. However, there is convincing evidence that structural and functional abnormalities in the basal ganglia, in cortico-striato-thalamo-cortical circuits, and some cortical areas including medial frontal regions and the prefrontal cortex as well as hyperactivity of the dopaminergic system are key findings. Conventional therapeutic approaches in addition to counseling comprise behavioral treatment, particularly habit reversal therapy, oral pharmacotherapy (antipsychotic medication, alpha-2-agonists) and botulinum toxin injections. In treatment-refractory Tourette syndrome, deep brain stimulation, particularly of the internal segment of the globus pallidus, is an option for a small minority of patients. Based on pathophysiological considerations, non-invasive brain stimulation might be a suitable alternative. Repetitive transcranial magnetic stimulation appears particularly attractive. It can lead to longer-lasting alterations of excitability and connectivity in cortical networks and inter-connected regions including the basal ganglia through the induction of neural plasticity. Stimulation of the primary motor and premotor cortex has so far not been shown to be clinically effective. Some studies, though, suggest that the supplementary motor area or the temporo-parietal junction might be more appropriate targets. In this manuscript, we will review the evidence for the usefulness of repetitive transcranial magnetic stimulation and transcranial electric stimulation as treatment options in Tourette syndrome. Based on pathophysiological considerations we will discuss the rational for other approaches of non-invasive brain stimulation including state informed repetitive transcranial magnetic stimulation.

## Clinical Phenomena and Previous Directions of Research and Therapy

Gilles de la Tourette syndrome (GTS) is a multifaceted neuropsychiatric disorder typically commencing in childhood. It is characterized by motor and phonic tics lasting for at least one year with onset before the age of 18 years (1). Tics typically start around the age of 6 (2), mainly as simple motor tics affecting the face (2), followed by phonic tics a few years later (3). Repertoire and severity of both motor and phonic tics vary widely encompassing for instance activation of single muscles or small muscle groups leading to discreet movements such as e.g., blinking, screwing up the eyes or eye-rolling, brief sounds like sniffing, throat clearing, grunting or single syllables, but also complex movements like squatting, body turning or twisting and the utterance of words or sentences. Tics resemble physiological movements and actions (4). However, they typically exhibit a repetitive pattern and appear temporally and situationally misplaced (5). Tic repertoire and severity fluctuate over time (“waxing and waning”) (2). In most cases, patients report various sensory phenomena preceding tics (“premonitory urges”) (6), which are relieved, at least transiently, by tic execution (2). Stress and focusing on tics lead to an increase of symptoms, whereas distraction ameliorates them (7, 8). Some 60% of GTS patients also have attention deficit hyperactivity disorder (ADHD) (9) and about 40 % obsessive-compulsive disorder (OCD) (10). After a symptom peak during pre-puberty, symptoms subside or improve considerably toward the end of the second decade in most patients (2). Still, there remains a fraction of patients who continue to have troublesome tics in adulthood.

The pathophysiology of GTS, particularly the nature of tics, is still incompletely understood and a matter of debate (11–13). However, there is increasing evidence that structural and functional abnormalities in the basal ganglia, e.g., a volume reduction of the striatum (14, 15), in cortico-striato-thalamo-cortical circuits (16, 17), and in some cortical areas including medial frontal regions and the prefrontal cortex (15, 18) as well as hyperactivity of the dopamine system (19–21) are key findings.

As outlined above, in most GTS cases, the clinical course is benign with remission or substantial improvement in early adulthood (2). In many patients, apart from counseling no specific therapy is needed. If symptoms are troublesome, therapeutic approaches encompass behavioral cognitive therapy (22), particularly habit reversal therapy (23), oral pharmacotherapy (antipsychotic medication, alpha-2-agonists) (24, 25), and botulinum toxin injections (26). In some patients, these measures are not sufficiently helpful, or cause intolerable side effects. In treatment-refractory GTS, deep brain stimulation (DBS), particularly of the internal segment of the globus pallidus and thalamic nuclei, is an option for a small minority of patients (27). DBS is an invasive procedure with possible untoward side effects, particularly dysarthria or infections related to the implanted device (28), which is particularly problematic in a potentially transient disorder like GTS. Therefore, alternative non-invasive therapeutic brain stimulation options would be welcome.

## What Is Neurostimulation?

Neurostimulation comprises interventions aiming at modulating neural networks using electric stimulation in the form of DBS, electroconvulsive therapy, transcranial magnetic stimulation (TMS), transcranial electrical current stimulation, and vagus nerve stimulation (29). Methods being applied without surgical intervention such as electroconvulsive therapy, TMS, transcranial electrical current stimulation and transcutaneous vagus nerve stimulation are referred to as non-invasive brain stimulation (NIBS) and continue to gain importance both for experimental and therapeutic purposes. In this review, we will focus on TMS and transcranial electrical current stimulation.

## Repetitive Transcranial Magnetic Stimulation (rTMS) as a Treatment for GTS

Given limitations of behavioral interventions, pharmacological therapy, or stereotactic neurosurgery for the treatment of GTS with respect to efficacy or side effects (2, 30), alternative non-invasive brain stimulation approaches have been explored over the past 20 years. This is particularly true for transcranial brain stimulation comprising techniques directly or indirectly inducing electric currents within discrete cortical regions and interconnected brain networks (31). A distinction is made between transcranial electrical current stimulation and TMS. Transcranial electrical current stimulation uses direct electric currents applied to the scalp via electrodes (32), TMS induces electric currents in the brain through magnetic fields produced by electromagnetic coils positioned over the scalp (33). Whereas single or paired-pulse TMS allowing to measure the excitability and activity of motor cortical and interconnected areas is of great interest for studying the pathophysiology of GTS (34), repetitive TMS (rTMS) provides the opportunity of inducing effects outlasting the time of stimulation. This could lead to prolonged excitability changes in discrete brain regions, i.e., neuroplasticity (34), rendering it particular attractive for therapeutic interventions. Lower frequency rTMS around 1 Hz typically induces a net decrease of the excitability of the targeted area/network and higher frequencies cause a net increase (33). Importantly, excitability changes caused by rTMS can last for minutes up to hours (35). These effects are due to synaptic plasticity referring to the ability of synapses to alter synaptic transmission as a function of their activation patterns (36). Whereas short-term plasticity lasts for tens of milliseconds to a few minutes (36), long-term plasticity lasts for minutes and hours (37). Long-term plasticity forms the basis of long-lasting rTMS effects and represents a key mechanism of rehabilitation, learning and memory (38). It encompasses changes within membranes of synapses leading to enhanced (long term potentiation) or reduces (long term depression) synaptic transmission (37). Underlying mechanisms of rTMS are mediated predominantly by post synaptic glutamate receptors (37). Whereas α-amino-3-hydroxy-5-methyl-4-isoxazolepropionic acid (AMPA) receptors activated by glutamate allow an influx of sodium ions leading to depolarization, N-Methyl-D-Aspartat (NMDA) receptors are blocked by magnesium ions (37). However, in case of an extensive use of synapses comprising long lasting depolarization, magnesium ions release the NMDA receptors resulting in an influx of calcium ions. Subsequently, calcium influx entails numerous adaptive processes including phosphorylation processes of AMPA receptors increasing their activity (37).

In contrast to TMS, transcranial electrical current stimulation does not cause action potentials directly (39). It rather modulates neuronal transmembrane potentials increasing neural excitability at the anodal electrode and decreasing it at the cathodal electrode (40). Transcranial electrical current induced activation is thought to be mediated by decreased γ-aminobutyric acid (GABA) concentrations (41) as well as increased brain-derived neurotrophic factor (39), and glutamate and glutamine concentrations (42).

New rTMS protocols include theta burst stimulation (TBS). Whereas in traditional rTMS, stimulation is defined predominantly by the firing frequency, e.g., 1 or 5 Hz, TBS is characterized by the firing pattern. Thus, three TMS pulses are applied with a high frequency (typically 50–100 Hz over 100–1,000 ms) (43). This burst of three stimuli is repeated every 200 ms, e.g., at a frequency of 5 Hz (43). TBS can either be delivered continuously for 40 s. (continuous TBS) resulting in a decrease of cortical excitability or intermittently as 2 s. trains repeated every 10 s. for a total of 190 s (intermittent TBS) leading to an increase of cortical excitability if applied over the primary motor cortex (M1) (43). Advantages of TBS encompass shorter application times and lower stimulation intensities compared to conventional rTMS (35), but variability of effects is higher (44).

When considering rTMS in GTS, several general questions arise. First, should based on alterations of defined neuronal circuits, inhibitory or excitatory protocols be used? Second, which brain regions are promising targets? Third, can findings of neurostimulation effects in healthy controls be extrapolated to the group of GTS patients, i.e., is the reactivity of the GTS brain to neurostimulation similar to that of healthy controls? Forth, which is related to the third question, is non-invasive stimulation timed and targeted to a defined brain's state at the time of stimulation sensible and feasible?

### Circuit Based rTMS

Regarding motor disorders one possible way to decide which rTMS protocol should be used are measurements of the excitability of the motor system using TMS with the notion to influence motor output.

#### Corticospinal and Short-Range Intracortical Sensorimotor Circuits

In adult GTS patients, RMT was shown to be normal (45–51). In children with GTS, results were more variable, showing either normal (45) or increased RMT (52). AMT seems normal in GTS patients (45, 47, 51). Input output curves are also normal in GTS (48, 49) or even shallower compared to healthy controls (51, 52). Thus, net corticospinal excitability at rest seems to be rather normal or decreased in GTS. It cannot be used as an argument for either excitatory or inhibitory rTMS.

During volitional movement preparation, the gain in motor cortex excitability is reduced in GTS (48, 52, 53). This appears to be associated with increased GABA levels in the supplementary motor areas (SMA) (54). It might be argued that the normal or even decreased motor cortex excitability in GTS reflects inhibitory processes related to tic inhibition. This is corroborated by a study showing a decrease in motor cortex excitability during active tic suppression (55). It is thus likely that reduced excitability during motor preparation is an adaptation of the motor system to compensate for tic related overactivation. One way to strengthen such adaptation might be to use inhibitory rTMS attenuating overactivity caused by tics.

Silent period defines a temporary reduction of EMG activity in tonically pre-activated muscles caused by supra-threshold TMS pulses (56). Such stimuli can target different structures of the motor system such as peripheral nerves, the cervico-medullary junctions or the motor cortex (55). Its duration depends on different elements of the motor system between the stimulation site and EMG recording. Cortical silent period refers to the EMG activity reduction of tonically pre-activated muscles due to TMS given to the contralateral motor cortex (57). It is mediated by GABA-B receptors (58). Its duration is highly variable, increasing with stimulation intensity (59) and exhibiting a high interindividual variability (60). Whereas the first 50 ms of the CSP seem to be caused by spinal inhibitory mechanisms, the main part (50 up to 300 ms) is due to various cortical inhibitory processes (61). Thus, CSP serves as a composite measure of cortical inhibition (62). Interestingly, CSP is shortened in GTS patients providing evidence for impairment in cortical inhibition (46, 63, 64). However, some studies did not find differences in CSP between GTS patients and healthy controls (51, 65).

Using a paired-pulse paradigm measuring short-interval intracortical excitability (35), where sub-threshold conditioning pulses modulate the effects of a subsequent, supra-threshold test pulse causing short-interval intracortical inhibition (SICI) at inter stimulus intervals of 1–5 ms and facilitation at intervals of 7–20 ms referred to as intracortical facilitation (35), SICI was shown to be reduced in GTS (47, 48, 51, 64, 65) whereas intracortical facilitation seems to be normal (45, 47, 48, 64). Given that SICI is predominantly mediated by GABA-A interneurons (66), these findings suggest a potential reductions of synaptic GABA-A activity in GTS.

Another way of examining intracortical excitability is to analyze short-latency afferent inhibition (SAI). It is measured by delivering electrical stimulation to the median nerve at the hand or the wrist 20–25 ms prior to a TMS pulse applied to the hand area of the motor cortex (67). This results in a reduction of the subsequent EMG amplitude (67), the basis of which is short-latency afferent input from peripheral receptors via the somatosensory cortex to the motor cortex (68). In patients with implanted cervical epidural electrodes, it could be shown that EMG amplitude reduction is due to reduced corticospinal output (67). Since these effects only emerge following TMS activating corticospinal neurons via their synapses and not following transcranial electrical stimulation directly activating corticospinal neurons at their axons, it becomes evident that SAI is mediated by intracortical inhibitory projections in sensorimotor cortical areas (67, 69). In GTS, SAI was found to be decreased (47, 70).

Taken together, as regards sensorimotor circuit abnormalities in GTS, there are no unequivocal and consistent findings. Even though some studies showed deficits in intracortical inhibition in GTS suggesting the use of inhibitory low-frequency rTMS applied to sensorimotor cortical regions, these findings are not undisputed. For instance, inhibition of the motor cortex using 1 Hz rTMS was clinically ineffective (71, 72). Therefore, rational circuit based rTMS application is currently not feasible.

#### Long-Range Frontal and Basal-Ganglia-Thalamo-Cortical Circuits and Inhibitory Control

Arguments to use inhibitory TMS protocols have also been derived from a number of studies suggesting deficits in inhibitory control in GTS mediated by the frontal cortex and basal-ganglia-thalamo-cortical circuits (71–80). However, it is important to note that findings suggesting impairments in inhibitory control are not undisputed. There are also studies showing no difference (15, 81–83), sometimes depending on the difficulty to inhibit a response (12, 84), or even enhanced performance (85–87) in inhibitory tasks in GTS. Also, the notion of a general impairment of inhibitory functions in GTS is contentious given the ability of these patients to actively suppress their tics, at least for certain periods of time (88). However, a meta-analysis confirmed a small to medium deficit of inhibitory control in GTS patients (89).

Of note, inhibitory control encompasses different sub-processes including automatic/habitual and volitional/goal-directed inhibition (90, 91). In contrast to automatic inhibition, volitional inhibition occurs in conditions demanding active suppression of actions, e.g., in Go/Nogo or Stop signal tasks, as well as during active suppression of tics. Furthermore, volitional inhibition can be subdivided into reactive inhibition (stop of a response instructed by a stop signal) and proactive inhibition (preparation for stopping a response due to a potentially upcoming stop signal) (92). Previous studies in GTS have largely focused on proactive and reactive inhibition. However, since the ability of GTS patients to actively suppress their tics suggests normal volitional inhibitory processes, it is of great interest to also examine processes of automatic inhibition. Rawji et al. examined volitional and automatic inhibition in GTS (93). Whereas volitional proactive and reactive inhibition were normal in GTS, automatic inhibition tested in a masked priming task differed from healthy controls, suggesting impairments in automatic inhibition in these patients (93). In contrast, Stenner et al. found strong automatic inhibition both in GTS and healthy controls without a significant group difference (94). Although the neural substrate for such impaired automatic inhibition is currently unclear, it is likely that cortico-subcortical networks including the medial prefrontal cortex and the striatum play a role (95, 96). These findings imply that inhibitory rTMS protocols reducing the strength of excitatory and boosting the strength of inhibitory circuits in fronto-striatal networks possibly leading to increased automatic inhibition could reduce tics since weakened automatic inhibition might be responsible for the occurrence of tics.

### Target Region Based Non-invasive Brain Stimulation

Given that the strength of the magnetic field and correspondingly also the strength of the electric current induced decrease with increasing distance from the coil, significant direct activation of cerebral structures is feasible up to 2–3 cm beneath the surface of the brain. However, as outlined above, inhibitory processes in cortico-striato-thalamo-cortical, fronto-striatal and basal ganglia circuits seem to be particularly relevant in GTS. Subcortical regions including the basal ganglia though cannot directly be activated by rTMS. However, indirect stimulation of subcortical regions is feasible due to neural pathways connecting cortical with subcortical regions (97). Thus, a suitable target for rTMS should be located in cortical regions shown to be relevant for the pathophysiology of GTS and connected to cortico-striato-thalamo-cortical circuits.

To ensure that the coil is placed correctly on the scalp, different strategies can be used. Which one to apply depends on the level of accuracy and replicability needed. One straightforward way to find the desired target area is to identify it on the basis of measurable output. For example, this can be achieved by registering the occurrence of phosphenes following stimulation of the visual cortex (98, 99), or, of course, recording of motor evoked potentials after M1 stimulation. Alternatively, the international 10–20 EEG-system can be used for localization on the basis of anatomical landmarks (100), obviously disregarding interindividual differences. For higher precision, a frameless stereotaxic neuronavigation system can be utilized. Using this method, based on MRI scans both the surface of the head and of the brain is calculated. In the next step, three light emitting diodes are attached to the head as well as to the stimulation coil, which are captured by three cameras interrelating them spatially. This setup makes it possible to find and to monitor the correct area of the cortex being stimulated without fixating the head (101).

**Figure 1:**
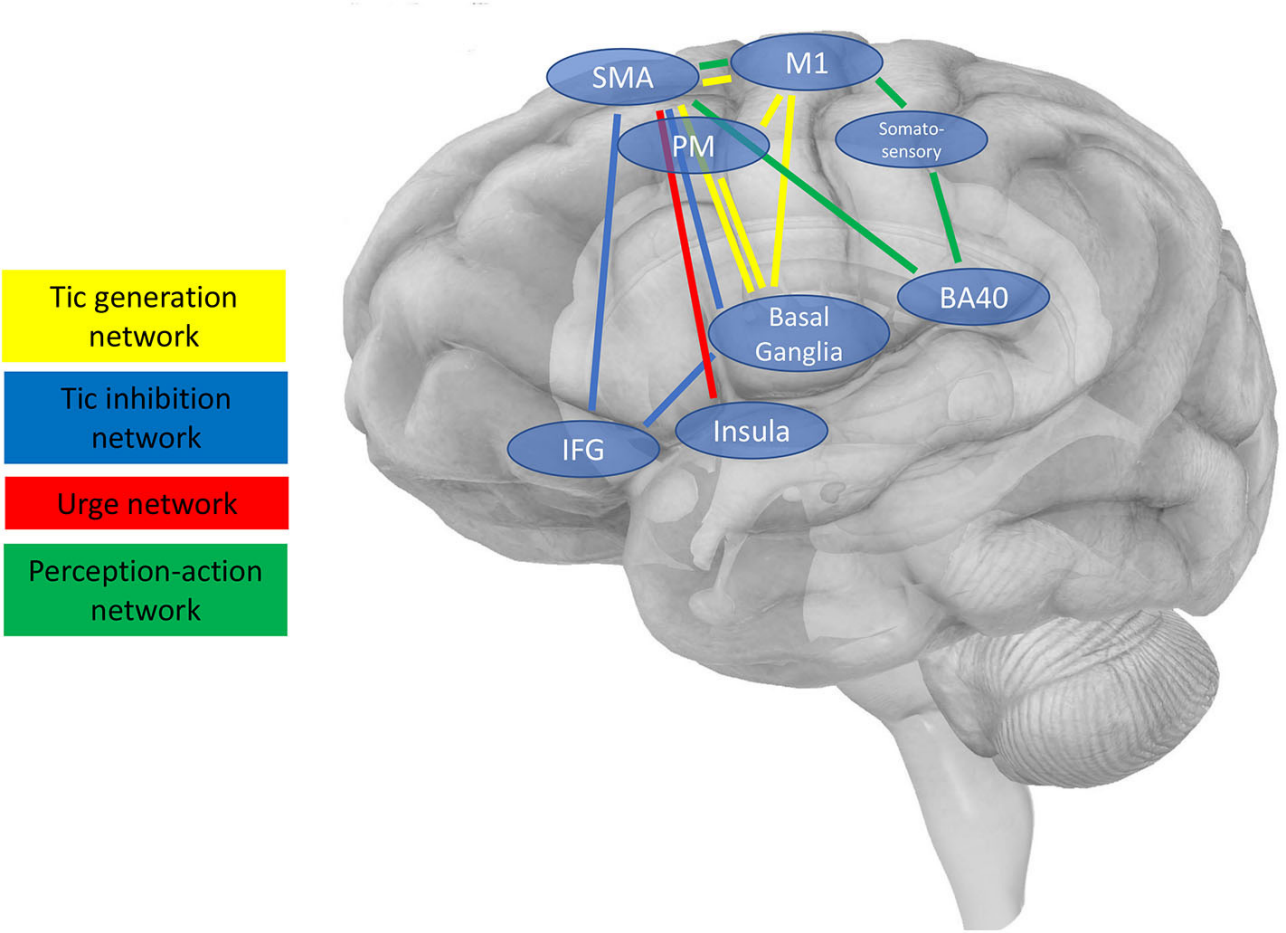
Schematic presentation of cortical regions and interconnected brain networks, which are possible targets for NIBS. Four main networks are shown as described in the main text (3.2.), i.e., (i) the tic generation network (shown in yellow), (ii) the tic inhibition network (blue), (iii) the urge network (red), and (iv) the network engaged in perception-action integration considered to be relevant for the occurrence of tics based on pathophysiological considerations.

In general, cortical target regions for NIBS, i.e., accessible nodes of larger neuronal networks, can be chosen with different aims, for instance (i) to directly reduce tic production, (ii) to increase voluntary tic control, i.e., foster tic inhibition enabling patients to suppress their tics more efficiently, (iii) to reduce premonitory urges, or (iv) to influence processes considered relevant for the occurrence of tics based on pathophysiological considerations, e.g., perception-action processes. Such targets and their connected networks are illustrated in Figure 1 and are described in the following section. Table 1 provides an overview of studies available addressing these targets by means of brain stimulation.

**Table 1:** Non-invasive brain stimulation parameters used in Tourette patients.

**References**	**Design, population characteristics**	**Stimulation details**	**Stimulation site**	**Results/Outcome measures**
Munchau et al. (71)	rTMS, RCT, single blinded, crossover, *N* = 16 GTS (adults) Comorbidities: *N* = 7 OCD	1,200 pulses in 1 session per day for 2 days, 1 Hz, 80% AMT 2 week interval between sites	Left motor cortex Left premotor cortex Sham	No significant clinical improvement in: MOVES HDS-D
Orth et al. (72)	rTMS, RCT, single blinded, crossover, *N* = 5 GTS (adults) Comorbidities: *N* = 2 ADHD	1,800 pulses in 1 session per day for 2 days, 1Hz, 80% AMT 4 week interval between sites	Left + right premotor cortex Left premotor cortex + right premotor cortex sham Right + left premotor cortex sham	No significant clinical improvement in: YGTSS MOVES MRVS
Mantovani et al. (76)	rTMS, open-label, *N* = 3 GTS, *N* = 5 OCD, *N* = 2 OCD+GTS (adults)	1,200 pulses divided in 4 sessions per day over 10 days, 1 Hz, 100% RMT	SMA (bilateral)	Significant clinical improvement in: YGTSS YBOCS HDRS-24 HARS-14 CGI SCL-90 BDI SAD SASS
Kwon et al. (78)	rTMS, open-label, *N* = 10 GTS (children 11.2 ± 2.0 years) Comorbidities: *N* = 3ADHD, *N* = 2 Depression, *N* = 1 OCD	1,200 pulses divided in 4 sessions per day for 10 days, 1 Hz, 100% RMT	SMA (bilateral)	Significant clinical improvement in: YGTSS CGI No significant clinical improvement in: Conner's ADHD Scale K-ARS CDI STAI Computerized ADS
Le et al. (79)	rTMS, open-label, *N* = 25 GTS (children 10.6 ± 2.2 years)	1,200 pulses divided in 20 sessions over 20 days, 1 Hz, 110% RMT	SMA (bilateral)	Significant clinical improvement in YGTSS CGI SNAP IV CDI SCAS
Landeros-Weisenberger et al. (102)	rTMS, RCT (phase 1)/open-label (phase 2), *N* = 20 GTS (adults)	Phase 1: 1,800 pulses in 1 session per day over 15 days, 1 Hz, 110% RMT Phase 2: 1,800 pulses in 1 session per day over 30 days, 1 Hz, 110% RMT	SMA (bilateral)	Phase 1: No significant clinical improvement in YGTSS YBOCS PUTS ASRS Phase 2: Significant clinical improvement in YGTSS
Mrakic-Sposta et al. (103)	tDCS, RCT, single blinded, crossover, *N* = 2 GTS (adults)	2 sessions a day: 2 mA for 15 min, five consecutive days 2 weeks interval between sites	Left premotor cortex Sham	Significant clinical improvement in YGTSS VAS for general well-being
Carvalho et al. (104)	tDCS, open-label, *N* = 1 GTS (boy, 16 years)	1 sessions a day: 1.4 mA for 30 min, over 10 days	Pre-SMA (bilateral)	Significant clinical improvement in YGTSS
Eapen et al. (105)	tDCS, double blinded, crossover, *N* = 2 GTS (adults)	1 session a day: 2.4 mA for 20 min, three times a week, over 6 weeks three weeks active cathodal followed by sham or vice versa	SMA (bilateral) Sham	Significant clinical improvement in ATQ PUTS
Dyke et al. (106)	tDCS, single blinded, crossover, *N* = 10 GTS (adults)	1 session: 4.5 mA for 20 min and 1 session sham 1 week interval between sessions	SMA (bilateral) Sham	Significant clinical improvement in YGTSS MRVS
Wu et al. (107)	cTBS, double blinded, *N* = 12 GTS (children and adults)	2,400 pulses per day over 2 days, 90% RMT, 30 Hz	SMA (bilateral) Sham	No significant clinical improvement in YGTSS

*ADHD, Attention deficit hyperactivity disorder; AMT, Active motor threshold; ASRS, Adult ADHD self-report scale; ATQ, Adult tic questionnaire; BDI, Beck depression inventory; CDI, Children's' depression inventory; CGI, Clinical global impression; Computerized ADS, Computerized ADHD diagnostic system; cTBS, continuous theta burst stimulation; GTS, Gilles de la Tourette syndrome; HARS-14, Hamilton anxiety rating scale; HDRS-24, Hamilton depression rating scale; HDS-D, Hospital anxiety and depression scale; K-ARS, Korean ADHD rating scale; mA, Milliampere; MOVES, Motor tic, obsessions and compulsions, vocal tic evaluation survey; MRVS, Modified Rush video scale; OCD, Obsessive-compulsive disorder; PUTS, Premonitory Urge for Tic Scale; RCT, Randomized controlled trial; RMT, Resting motor threshold; rTMS, repetitive transcranial magnetic stimulation; SAD, Scale for auto-evaluation of depression; SASS, Social adaptation self-evaluation scale; SCAS, Spence children‘s anxiety scale; SCL-90, Symptoms check list; SMA, Supplementary motor area; SNAP IV, Swanson, Nolan and Pelham rating scale; STAI, State/trait anxiety inventory; tDCS, transcranial direct current stimulation; VAS, Visual analog scale; YBOCS, Yale-Brown obsessive-compulsive scale; YGTSS, Yale Global tic severity scale*.

#### Tic Generation Network - Motor, Premotor Cortex, Supplementary Motor Area

Because imaging studies revealed that during the occurrence of tics metabolism was increased in the motor and premotor cortex indicating increased activity in these brain areas (108–110) and given that these areas are tightly connected to the basal ganglia, they represent attractive targets. In a study using 1,200 stimuli of 1 Hz left premotor or motor cortex rTMS at low intensity, i.e., 80% AMT, no clinical effects on tics could be demonstrated (71). In a follow-up single blinded placebo-controlled cross-over study, the number of stimuli was increased to 1,800 and applied to the premotor cortex of both hemispheres in turn (72). This study also failed to show any clinically meaningful effects. It could be argued that the intensities used in these studies were too low to cause clinically apparent changes of cortical network activity. However, particularly the premotor cortex is located close to the surface, so that even low TMS intensities are expected to lead to effective activation of this area. Thus, it appears plausible to assume that the motor and premotor cortex are not the optimal target region for rTMS in GTS.

The supplementary motor area (SMA) might be an alternative target. Since it is characterized by a high interconnection with the basal ganglia (111–114), it can be considered a central hub within the cortico-striato-thalamo-cortical circuits. The central role of the SMA in tic generation is underpinned by increased metabolic activity (108) and increased GABA levels correlating with tic severity (54) in this region. Additionally, comparison of spatiotemporal patterns of motor cortex co-activation during tic execution in GTS and tic-like movements in healthy controls revealed differences in the SMA (115). In line with this, event-related fMRI, carried out while GTS patients exhibited a variety of spontaneous tics, revealed the SMA to be active just prior to tic onset (116–118). Also, enhanced structural connectivity has been demonstrated in white matter pathways connecting the striatum and thalamus with M1 and SMA, which positively correlated with tic severity (17). Using real-time fMRI data, Hampson et al. showed that biofeedback-induced changes in SMA activity in healthy controls resulted in changes in resting state functional connectivity of SMA and subcortical regions (119). This provides strong evidence for activity changes in the SMA to reduce the influence of subcortical loops on the SMA, which are known to be altered in GTS. Building on that, Sukhodolsky et al. executed real-time fMRI biofeedback addressing SMA activity in GTS. Tic severity assessed using the Yale Global Tic Severity Scale significantly decreased after biofeedback intervention, which was not the case in the control group receiving sham intervention (120).

In addition to tic-related activity in the SMA, in GTS patients, activity of this region has also been shown to be abnormal during defined motor and cognitive control tasks. For instance, in a Go/NoGo reaction time task using event-related fMRI, there was reduced SMA activation in the Go conditions (121). In addition, reactive inhibition tested in a stop-signal reaction time task was normal in GTS, but the brain activation pattern during this task differed from healthy controls (83). Whereas right pre-SMA activation correlated with successful stopping in healthy controls in keeping with previously reported stop signal task-related activations (122, 123) and a general role of the pre-SMA/SMA in inhibitory control (124–127), this was not the case in GTS. Instead, in the activation contrast “successful stopping” vs. “Go,” there was a positive correlation between tic severity and right SMA-proper, but not pre-SMA activation, in GTS (83). Magnetoencephalography during a self-paced finger movement task showed stronger SMA-M1 coupling in GTS (128).

Plasticity in SMA-M1 circuits seems to be unchanged in GTS. Thus, in an SMA-M1 paired associative stimulation (PAS) protocol plasticity in SMA-M1 was normal in GTS (50). This suggests that the SMA is not primarily implicated in the (learning associated) formation of tics or the propensity to develop tics probably predominantly determined by the basal ganglia (5). It does not generally question the role of the SMA in the pathophysiology of tics though because the SMA might be particularly relevant with respect to tic occurrence and the inner structure of tics, given that the SMA is physiologically engaged in the preparation and temporal organization of self-initiated movement (129).

Given the prominent role of the SMA in tic generation, abnormal SMA activity in GTS during different motor tasks and findings of structural and functional abnormalities of the SMA, as well as its strong interconnection within the motor circuit, the SMA might be considered an attractive target for rTMS, primarily with the aim to reduce tic-related over-activity of this area. However, it has to be borne in mind that the SMA is located at a distance from the skull at the median surface of the brain in the interhemispheric cleft anterior to the leg area of M1. Therefore, high TMS intensities are needed for effective SMA activation likely leading to co-activation of adjacent areas, particularly the motor, and premotor cortex limiting the specificity of stimulation effects.

So far, data on clinical efficacy of rTMS given to the SMA is limited. In 2005, Mantovani applied 1 Hz rTMS over this region in an uncontrolled open label study. The rTMS coil was oriented along the sagittal midline with the handle pointing toward the occiput thus inducing a posterior-anterior current flow in the SMA. One thousand two hundredth stimuli per day over 10 days using an intensity of 100% of RMT led to a significant improvement of clinical global impression in the entire sample of 10 adult patients with GTS and/or OCD. Parallel to these findings, there was an increase of RMT. Clinical improvement was still present in follow-up examinations 3 month later (76). Another study using the same protocol found a reduction of tics in 10 children with GTS (78). In children, applying 1 Hz rTMS over the SMA with a posterior-anterior current flow using an intensity of 110% of RMT positive effects, i.e., a significant reduction both of Yale Global Tic Severity Scale and clinical global impression as well as a significant decrease of ADHD symptoms measured by Swanson, Nolan and Pelham Rating Scale, version 4, was found. Importantly, these effects have been shown to last for up to 6 months (79). Thus, 1 Hz rTMS targeting the SMA might be a promising approach for conducting controlled trials in GTS. The only controlled trial, however, failed to show any effect of 1Hz rTMS intervention over the SMA (102). Again, current flow was posterior-anterior. In line with this, eight sessions of continuous TBS (30 Hz, 90% RMT) delivered over 2 days did not show significant effects in a randomized, double-blind trial with 12 GTS patients (107).

Although there is some data suggesting that SMA stimulation might be promising in GTS, caution is required when interpreting these data. As mentioned above, due to its location at a distance from the surface, the SMA is not a straightforward rTMS target limiting the validity and specificity of reported findings. In addition, follow-up periods after rTMS interventions were short in some studies and patient numbers limited. These limitations are very relevant given that tics naturally fluctuate considerably, particularly in children and adolescents. Also, clinical outcome measures were subjective and assessment mostly open-label or single blinded; only two studied were double blinded.

#### Tic Inhibition Network- Inferior Frontal Gyrus (IFG)

Tics can be suppressed voluntarily (130). Thus, one might argue that addressing this core feature of GTS might be a fruitful approach. Voluntary tic control seems to be located in discrete cerebral regions (83). Using fMRI, Ganos et al. showed the inferior frontal gyrus (IFG) to be more active in a state of voluntary tic inhibition compared to a free ticcing condition (83). Additionally, IFG activity during tic inhibition was positively correlated with the ability to inhibit tics (83). Furthermore, fMRI studies suggest an increased activity in the caudate nucleus together with a decreased activity of the thalamus, putamen and globus pallidus (109), an increased activity of the left anterior cingulate cortex (131) and increased activity in the dorsal anterior cingulate cortex and associated limbic areas (132) during tic inhibition. Given not only an increased activity during voluntary tic suppression (83), but also a positive correlation of this activity with the ability of tic suppression (83), it seems reasonable to boost IFG activity by means of rTMS. To this end, high-frequency stimulation inducing facilitation might be useful. The right inferior frontal gyrus has been suggested to play a more general role in “inhibitory control” (133). Interestingly, it has been shown that using TBS protocols (134), processes in the right inferior frontal gyrus can be modulated to increase and decrease inhibitory control. Therefore, aside rTMS protocols also TBS protocols may be useful to modulate inferior frontal gyrus activity in GTS.

#### Urge Network–Anterior Insula

Given that urges preceding tics might be a driving force for the occurrence of tics (6, 88), stimulation of “urge” areas, i.e., regions associated with the generation of urges, with inhibitory rTMS might lead to an attenuation of both urges and tics. In addition to the SMA and the inferior parietal cortex (BA40), the insula has, as pointed out above, also been implicated in the generation of urges in GTS. More specifically, using resting-state fMRI, the right anterior insula showed higher connectivity with cortico-striato-thalamocortical nodes and functional connectivity between the right anterior insula and bilateral SMA correlated with urge severity in adult GTS patients (135). Also, using whole-brain analysis of cortical gray matter, thickness was reduced in the insula and sensorimotor cortex in children and young adults with GTS compared to healthy controls (53). It was also demonstrated that urges were inversely associated with gray matter thickness measurements in these areas (53). Given these data, the anterior insula also appears as a region of interest for NIBS. However, it should also be mentioned that targeting the insula requires coil placement fronto-laterally, which may lead to direct activation of the masseter muscle causing discomfort.

#### Perception-Action Integration Network–Inferior Parietal Cortex (BA 40)

The presence of urges and also hypersensitivity to certain sensory stimuli (136), e.g., increased distractibility and distress by tactile stimuli (136, 137), suggests that somatosensory processing is altered in GTS (138). The integration of sensory information with motor planning and execution seems to be impaired. This may be related to structural abnormalities including thinning of the somatosensory cortex in adolescent GTS (139) and white matter abnormalities underneath the primary somatosensory cortex (BA 3a) in adult GTS patients (140). This notion is supported by fMRI-based functional connectivity analyses showing reduced connectivity in long-range fronto-parietal networks (141, 142). In addition, in periods preceding tics, increased activation has been shown in the inferior parietal cortex (BA 40) (116), an important relay of perceptual processing (143, 144). In addition, although basic perception of somatosensory stimuli did not differ between GTS and healthy controls (145) a number of studies document functional and structural abnormalities in somatosensory-motor pathways in these patients (47, 139, 140, 146–148). Finally, sensorimotor integration has been shown to be altered in GTS. For instance, in grip force experiments, GTS patients used higher grip forces to hold an object with defined weight than healthy controls (149, 150). Also, short afferent inhibition addressing sensorimotor integration was found to be reduced in GTS (47, 147). Given that BA 40 is activated before the occurrence of a tic (116) and given its role as a hub of perception-action integration also relevant during inhibitory control (144, 151–153), it is likely that BA 40 is involved in abnormal sensorimotor processes in GTS.

Recent findings derived from behavioral experiments comprising visuomotor stimulus-response tasks confirmed alterations of central processing of perceptions and actions in GTS. Against the background of clinical findings suggesting a strong interaction between motor (i.e., tics) and perceptional (e.g., premonitory urges) processes (88), Kleimaker et al. (13) examined perception-action processes in the context of the Theory of Event Coding (TEC) (154). Presenting a general framework for the cognitive basis of perception and action paying particular attention to their dependency, TEC considers sensory stimuli and motor actions to be bound and stored together in so-called “event files” (154). That means that sensory consequences emerging from an action are linked to this action and vice versa. In this study, a previously established visuomotor task was used (155), allowing to test the strength of perception-action bindings directly. This study yielded robust evidence for stronger perception-action bindings in these patients (13). Brain electromagnetic topography showed these effects to originate from BA 40 (13). In line with this, Petruo et al. (12) carried out an unimodal vs. bimodal visual/acoustic Go/NoGo paradigm in adolescents with GTS. They showed increased binding between bimodal stimuli and responses leading to increased costs of switching between responses instructed by bimodal and those instructed by unimodal stimuli. The neurophysiological data demonstrated that this was related to perception-action binding processes in the right BA 40 (12).

Thus, BA 40 should also be considered as a target area for non-invasive brain stimulation including rTMS. Inhibitory rTMS might be used to attenuate abnormally increased perception-action binding in GTS.

### Reactivity of the GTS Brain to Neurostimulation

When considering plasticity inducing protocols as a treatment in GTS, the principal questions arise as to whether brain responses in GTS patients are expected to be similar to healthy controls and whether the direction of changes can be extrapolated from established alterations in healthy controls. In this context, it is relevant to consider previous studies using plasticity inducing protocols in GTS. Long-term potentiation and long-term depression like plasticity can experimentally be induced in the motor cortex in humans using rTMS protocols including TBS, high frequency electrical stimulation, e.g., of the supraorbital nerve, and PAS (43, 156–158). Previous studies using intermittent and continuous TBS and inhibitory high-frequency electrical stimulation of the supraorbital nerve showed that plasticity is reduced in GTS patients (158, 159). Similarly, in a PAS protocol where peripheral electrical stimulation of the median nerve was coupled with TMS over M1, Brandt et al. demonstrated that there was no typical long-term depression-like effect in response to PAS in GTS (49). These studies thus suggest reduced plasticity in brainstem circuits (158, 159) and sensorimotor pathways (49). However, in another PAS study, using the same protocol as that of Brandt et al. (49), Martin-Rodriguez et al. reported long-term depression-like effects to be stronger in GTS compared to healthy controls (160). In addition to the high variability of stimulation effects in NIBS studies (157, 161, 162), these discrepant findings are probably also related to disease severity (50). Moreover, using a PAS protocol stimulating M1 bilaterally and the SMA, there was, as outlined above, a significant PAS effect in GTS that did not differ from healthy controls (50). This suggests that there is no global reduction of plasticity in neural networks in GTS, but apparently altered plasticity predominantly in sensorimotor and brainstem, but not in SMA-M1 circuits in GTS.

Taken together, it is conceivable that protocols typically inducing plastic changes in a certain direction in healthy controls will have different effects in GTS patients, because their brains' reactivity differs. In other words, potentially altered plasticity in GTS can limit the efficacy of rTMS interventions aiming to treat GTS patients or might even induce maladaptive plasticity. This needs to be borne in mind when planning and interpreting results of stimulation interventions in GTS patients.

Given that repeated rTMS interventions are needed to induce improvements in other diseases like Parkinson‘s disease (163), increasing the number of interventions might also compensate for a presumably altered reactivity of the GTS brain in these patients. In addition, in view of variable results of plasticity-inducing experiments in GTS, including only 10–15 subjects like in most previous studies (158, 159) might not be sufficient. Of note, when including 50 GTS patients, plasticity to TBS has been shown to be reduced in GTS (164).

### State Informed Brain Stimulation

The brains' state at the time of NIBS is a crucial factor for its effectiveness. Thus, application of a given stimulation protocol is likely to differ in a resting state compared to a ticcing state in GTS patients. Because it is not possible to experimentally induce a non-ticcing state in GTS patients other than by sedation/anesthesia, which, of course, profoundly affects the brains' responsiveness to interventions, it may be preferable to explicitly focus on states/periods when tics occur or are about to occur. A pre-requisite for such an approach would be biological markers reliably indicating that a tic is about to happen, so that an external intervention can be timed and targeted to obviate an imminent tic. This would represent a “closed-loop” approach. In “open loop systems,” any output does not influence the control action since the output is neither measured nor fed back (165). In “closed-loop systems” output is measured and fed back to adjust the control action, i.e., a bidirectional flow of signals in both responding and sensing direction is used to provide state adjusted interventions. A classic example of a “closed-loop” device is a cardiac pacemaker sensing heartbeat and adjusting stimuli to it (166).

In the field of neurostimulation, output measurement is more challenging since brain signals are much more complex than, for instance, P-wave signals of the heartbeat. So far, established devices for DBS, for instance, to treat patients with Parkinson‘s disease (167), dystonia (168), or GTS (27) are “open loop systems.” However, of late, there have been advances in the development of “closed-loop” approaches. There are promising sources of information such as local field potentials (LFP) representing the sum of extracellular electric activity of discrete populations of neurons measured by electrodes implanted into the brain (169). Importantly, LFP signals derived from cortical areas like the motor or visual cortex could be related to clinical parameters such as movement or visual perception (170). In 2013, “closed-loop” DBS treatment using LFPs has been implemented for advanced Parkinson‘s disease and shown to be effective (171). No such systems have been tested in GTS, mainly because no unequivocal neural signal as a marker of tics has been identified. Recently, though, recordings of oscillatory activity from the centro-median nucleus of the thalamus in patients receiving DBS revealed a low-frequency power (3–10 Hz) increase time-locked to the onset of tics but not during voluntary movements in GTS patients (172). Such activity might guide the development of “closed-loop” neuromodulation. However, a significant disadvantage of using DBS and LFP or oscillatory activity as “closed-loop” systems is the invasiveness of the procedure requiring neurosurgical intervention.

TMS capable of stimulating discrete cortical regions with a spatial resolution of centimeters and a temporal resolution of milliseconds (173) might be an alternative for non-invasive “closed-loop” stimulation. To “close the loop” non-invasively, EEG signals with high temporal resolution (174) as well as properties to reflect brain state changes caused by TMS (175) could be used. In fact, Zrenner et al. presented a real-time “closed-loop” system comprising a TMS/EEG set-up showing that a synchronization between EEG activity and TMS is possible and that a given brain state (defined on the basis of alpha band activity) affects both responsiveness to TMS and induced plasticity (176).

Regarding non-invasive “closed-loop” stimulation in GTS, the most relevant point relates to the question, which signal is accessible and could be used as a biological marker for tic-related activity to be targeted by rTMS. Using scalp EEG signals is problematic since there are only non-specific EEG changes (177) and motion artifacts caused by tics. Wearable motion capture devices recognizing tics might be a good option. Using a triaxial accelerometer placed on the patient‘s trunk, it might be possible to detect tics in the context of normal movements (178).

Alternatively, activity related to urges typically preceding tics could be targeted by rTMS. To this end, an online system capturing urge fluctuations, e.g., the urge monitor previously developed and validated in GTS, OCD and skin ticking disorder (88, 179, 180) coupled, for instance, with pupillometry could indicate brain states associated with high probability with the imminent occurrence of tics. Inhibitory rTMS applied to areas mediating the urge to tic, including the SMA or insular cortex (see above), might then disrupt the urge-tic cascade leading to the attenuation or “cancellation” of tics.

### Patient Assessment and Outcome Measures

The diagnosis of GTS should be made according to DSM-5 criteria (1). Additionally, somatic diseases as well as medication should be assessed since both might interfere with potential findings. Since GTS represents a complex neurodevelopmental neuropsychiatric disorder, patients need to be assessed accurately with a view to psychiatric comorbidities that might influence results of NIBS. To this end, the Mini International Neuropsychiatric Interview (M.I.N.I.) (181) should be carried out in any patient. For the assessment of typical comorbidities, particularly OCD and ADHD (see above), we recommend the Conners Adult ADHD Rating Scale (182) and the Yale Brown Obsessive Compulsive Scale (YBOCS) (183).

Measuring outcome in GTS is not straightforward since GTS patients' symptoms naturally fluctuate both within shorter and longer periods (2). Thus, performing clinical outcome measurements only shortly before and directly after rTMS interventions is not sufficient. They should instead be repeated several times before and after interventions to capture changes over and above natural fluctuations. This is also true for other behavioral measures, e.g., tic inhibition capacity.

Tic frequency and severity can be assessed using the Modified Rush Videotape Rating Scale (184). This protocol comprises a 10-min video recording of patients placed in front of a video camera in a quiet room. Two body views are recorded, full frontal body (far) and head and shoulders only (near) under two conditions: (1) relaxed with the examiner in the room and (2) relaxed with the patient alone in the room. Each video segment lasts 2.5 min. Only recordings with no examiner present are scored (5 min). Five domains are rated: number of body areas affected by tics, motor tic intensity, phonic tic intensity, frequency of motor tics, and frequency of phonic tics. On the basis of the Rush protocol tic count per minute can also be determined (13). Furthermore, symptom severity should be assessed using the Yale Global Tic Severity Scale (YGTSS) (185).

Premonitory urges can be assessed using a real-time monitoring system to quantify urge intensity in relation to tic frequency referred to as urge meter (179) as well as the Premonitory Urge for Tics Scale (PUTS) (186).

### Limitations of rTMS as a Treatment Option for Tourette Syndrome

Since neuroanatomical and neurophysiological alterations in GTS are still understood incompletely, it remains unclear whether rTMS induced plasticity differs between GTS patients and healthy subjects (see above), between different GTS patients or even within one subject, for instance during in a “ticcing-” compared to a “non-ticcing” -state. Thus, predicting the effectiveness of rTMS is currently not possible.

In addition to these and other open scientific questions outlined above for rTMS to produce clinically meaningful and sustained effects, it needs to be repeated on consecutive days for weeks greatly limiting its usefulness in clinical practice. Also, rTMS is so far only available in specialized research centers. Additionally, applying rTMS in GTS patients can be challenging due to tic related head and coil displacement. This can, at least in part, be tackled by coil holders and frames fixating the subjects' head.

### Transcranial Electric Stimulation as a Treatment for GTS

As mentioned above, transcranial electrical stimulation directly passes electric currents through the skull to the cortex (32). The most common method comprises constant low direct currents, referred to as transcranial direct current stimulation (tDCS). Furthermore, there are protocols using oscillatory stimulation (transcranial alternating current stimulation, transcranial random noise stimulation), or pulsed currents (transcranial pulsed current stimulation) (35). Here, we will focus on tDCS. Tonic and rather weak (1–2 mA) electrical currents crossing the scalp reach the brain surface with a loss of ~50% of amperage (187, 188). Focal, prolonged but reversible alterations (i.e., enhancement or reduction) of cortical excitability can be induced with tDCS (189–194). tDCS effects depend on current density (quotient of current strength and electrode size) (195) and stimulation duration (195). Increases of both current density (191, 196) and stimulation duration (189) are known to cause stronger effects. tDCS comprises two electrodes, one on the scalp over the target area and another elsewhere on the scalp or nearby. Polarity of the electrode applied over the target area defines whether enhancement (anodal stimulation) or reduction (cathodal stimulation) of excitability occurs (195). In contrast to TMS, direct activation of neurons by inducing action potentials is not feasible due to low amperage (195). Compared to TMS, tDCS-effects are less focal (197) and therefore probably less specific compared to TMS. Interestingly, in a dopamine transporter-overexpressing rat model, positive evidence has been found that anodal tDCS, applied to frontal regions, diminishes repetitive behaviors via the modulation of the striato-thalamo-cortical circuit, which is known to be critically hyperactive in GTS patients (198).

Similar to rTMS, tDCS research so far focused on inhibitory paradigms using cathodal tDCS-protocols targeting the motor cortex, the pre-SMA and SMA. In a pilot study, Mrakic-Sposta et al. applied cathodal tDCS for five consecutive days over the left motor cortex in two GTS patients (103). Additionally, sham stimulation was used for five consecutive days after a wash-out period of 2 weeks. Significant reduction of symptoms was shown after tDCS but not following sham stimulation. Carvalho et al. used cathodal tDCS over the pre-SMA in a 16 years-old male with complex and refractory motor and phonic tics (104) resulting in symptom reduction, which was still present 6 months after intervention. Eapen et al. applied sham-controlled cathodal tDCS to the SMA (105). Tic frequency, tic intensity and urge sensations were reduced significantly in two patients. However, in a case series study, Behler et al. (199) were not able to replicate these promising findings (127). The most recent study on tDCS in GTS included ten subjects examining immediate effect of 20 minutes of cathodal tDCS over the SMA compared to sham intervention (106). In order to obtain immediate effects, the Rush score (184) was determined directly prior to and after tDCS. Furthermore, motor cortex excitability was measured using single-pulse TMS. Rush scores were significantly lower after tDCS in comparison to sham intervention. However, there was no significant change of motor cortex excitability.

Summing up, there is tentative but very limited evidence that cathodal tDCS applied to the SMA and probably also the motor cortex might be effective in GTS.

## Summary

Given considerable side effects and therapy refractoriness using conventional therapy, new therapeutic approaches are needed for patients with GTS. In view of mounting insights into underlying processes related to the occurrence and control of tics on a neurophysiological and neuroanatomical level, neurostimulation seems promising and feasible. Given a fluctuating and often benign clinical course NIBS, particularly rTMS, intermittently used during times of tic exacerbation might become an attractive tool. Although direct effects of rTMS stimulation are restricted to cortical regions, activity in subcortical structures including the basal ganglia can be modulated indirectly through cortico-subcortical pathways. The SMA, inferior parietal cortex (BA 40) and the insula are potentially interesting target regions. So far, evidence for clinical efficacy of rTMS is very limited. Further studies including a higher number of patients, longer follow-up periods and using objective outcome measures including neurophysiological markers are required. Ideally, rTMS should be carried out using “closed-loop” setups. However, development of these systems is in its infancy and much more needs to be learned about urge- and tic-related brain processes and corresponding neurophysiological markers. In addition to rTMS, tDCS represents an interesting NIBS option.

## Author Contributions

MK and AK: writing of the first draft. AW, LC, CB, TB, and AM: review and critique. All authors contributed to the article and approved the submitted version.

## Conflict of Interest

The authors declare that the research was conducted in the absence of any commercial or financial relationships that could be construed as a potential conflict of interest.

## Abbreviations

AMT: active motor threshold
ADHD: attention deficit hyperactivity disorder
CSP: cortical silent period
DBS: deep brain stimulation; Gilles de la Tourette syndrome
IFG: inferior frontal gyrus
LFP: local field potential
M1: primary motor cortex
NIBS: non-invasive brain stimulation
OCD: obsessive-compulsive disorder
PAS: paired associative stimulation
RMT: resting motor threshold
rTMS: repetitive transcranial magnetic stimulation
SAI: short afferent inhibition
SICI: short interval intracortical inhibition
SMA: supplementary motor area
TBS: theta burst stimulation
tDCS: transcranial direct current stimulation
TEC: Theory of Event Coding
TMS: transcranial magnetic stimulation.

## References

[1] American Psychiatric Association Diagnostic and Statistical Manual of Mental Disorders. Fifth Edition American Psychiatric Association (2013). 10.1176/appi.books.9780890425596

[2] Leckman JF. Tourette's syndrome. Lancet. (2002) 360:1577–86. 10.1016/S0140-6736(02)11526-1 12443611

[3] Swain JE Leckman JF. Tourette syndrome and tic disorders: overview and practical guide to diagnosis and treatment. Psychiatry. (2005) 2:26–36. 21152158 PMC3000195

[4] Paszek J Pollok B Biermann-Ruben K Müller-Vahl K Roessner V Thomalla G. Is it a tic?–Twenty seconds to make a diagnosis. Mov Disord. (2010) 25:1106–8. 10.1002/mds.23053 20535828

[5] Ganos C Roessner V Münchau A. The functional anatomy of Gilles de la Tourette syndrome. Neurosci Biobehav Rev. (2013) 37:1050–62. 10.1016/j.neubiorev.2012.11.004 23237884

[6] Leckman JF Walker DE Cohen DJ. Premonitory urges in Tourette's syndrome. Am J Psychiatry. (1993) 150:98–102. 10.1176/ajp.150.1.98 8417589

[7] Brandt VC Lynn MT Obst M Brass M Münchau A. Visual feedback of own tics increases tic frequency in patients with Tourette's syndrome. Cogn Neurosci. (2015) 6:1–7. 10.1080/17588928.2014.954990 25185800

[8] Misirlisoy E Brandt V Ganos C Tübing J Münchau A Haggard P. The relation between attention and tic generation in Tourette syndrome. Neuropsychology. (2015) 29:658–65. 10.1037/neu0000161 25486384 PMC4484548

[9] Freeman RD Fast DK Burd L Kerbeshian J Robertson MM Sandor P. An international perspective on Tourette syndrome: selected findings from 3,500 individuals in 22 countries. Dev Med Child Neurol. (2000) 42:436–47. 10.1017/S0012162200000839 10972415

[10] Bloch MH Peterson BS Scahill L Otka J Katsovich L Zhang H. Adulthood outcome of tic and obsessive-compulsive symptom severity in children with Tourette syndrome. Arch Pediatr Adolesc Med. (2006) 160:65–9. 10.1001/archpedi.160.1.65 16389213 PMC2291298

[11] Beste C Münchau A. Tics and Tourette syndrome - surplus of actions rather than disorder? Mov Disord. (2018) 33:238–42. 10.1002/mds.27244 29278288

[12] Petruo V Bodmer B Brandt VC Baumung L Roessner V Münchau A. Altered perception-action binding modulates inhibitory control in Gilles de la Tourette syndrome. J Child Psychol Psychiatr. (2019) 60:953–62. 10.1111/jcpp.12938 29924402

[13] Kleimaker M Takacs A Conte G Onken R Verrel J Bäumer T. Increased perception-action binding in Tourette syndrome. Brain. (2020) 143:1934–45. 10.1093/brain/awaa111 32464659

[14] Peterson BS Thomas P Kane MJ Scahill L Zhang H Bronen R. Basal Ganglia volumes in patients with Gilles de la Tourette syndrome. Arch Gen Psychiatry. (2003) 60:415–24. 10.1001/archpsyc.60.4.415 12695320

[15] Müller-Vahl KR Kaufmann J Grosskreutz J Dengler R Emrich HM Peschel T. Prefrontal and anterior cingulate cortex abnormalities in Tourette Syndrome: evidence from voxel-based morphometry and magnetization transfer imaging. BMC Neurosci. (2009) 10:47. 10.1186/1471-2202-10-47 19435502 PMC2691409

[16] Makki MI Munian Govindan R Wilson BJ Behen ME Chugani HT. Altered fronto-striato-thalamic connectivity in children with tourette syndrome assessed with diffusion tensor MRI and probabilistic fiber tracking. J Child Neurol. (2009) 24:669–78. 10.1177/0883073808327838 19491113

[17] Worbe Y Marrakchi-Kacem L Lecomte S Valabregue R Poupon F Guevara P. Altered structural connectivity of cortico-striato-pallido-thalamic networks in Gilles de la Tourette syndrome. Brain. (2015) 138:472–82. 10.1093/brain/awu311 25392196 PMC4306818

[18] Fredericksen KA Cutting LE Kates WR Mostofsky SH Singer HS Cooper KL. Disproportionate increases of white matter in right frontal lobe in Tourette syndrome. Neurology. (2002) 58:85–9. 10.1212/WNL.58.1.85 11781410

[19] Singer HS Hahn IH Moran TH. Abnormal dopamine uptake sites in postmortem striatum from patients with Tourette's syndrome. Ann Neurol.. (1991) 30:558–62. 10.1002/ana.410300408 1838678

[20] Minzer K Lee O Hong JJ Singer HS. Increased prefrontal D2 protein in Tourette syndrome: a postmortem analysis of frontal cortex and striatum. J Neurol Sci. (2004) 219:55–61. 10.1016/j.jns.2003.12.006 15050438

[21] Palminteri S Lebreton M Worbe Y Hartmann A Lehéricy S Vidailhet M. Dopamine-dependent reinforcement of motor skill learning: evidence from Gilles de la Tourette syndrome. Brain. (2011) 134:2287–301. 10.1093/brain/awr147 21727098

[22] Piacentini J Woods DW Scahill L Wilhelm S Peterson AL Chang S. Behavior therapy for children with Tourette disorder: a randomized controlled trial. JAMA. (2010) 303:1929–37. 10.1001/jama.2010.607 20483969 PMC2993317

[23] Fründt O Woods D Ganos C. Behavioral therapy for Tourette syndrome and chronic tic disorders. Neurol Clin Pract. (2017) 7:148–56. 10.1212/CPJ.0000000000000348 29185535 PMC5669407

[24] Sallee F Kohegyi E Zhao J McQuade R Cox K Sanchez R. Randomized, double-blind, placebo-controlled trial demonstrates the efficacy and safety of oral aripiprazole for the treatment of tourette's disorder in children and adolescents. J Child Adolescent Psychopharmacol. (2017) 27:771–81. 10.1089/cap.2016.0026 28686474 PMC5689110

[25] Pringsheim T Holler-Managan Y Okun MS Jankovic J Piacentini J Cavanna AE. Comprehensive systematic review summary: treatment of tics in people with Tourette syndrome and chronic tic disorders. Neurology. (2019) 92:907–15. 10.1212/WNL.0000000000007467 31061209 PMC6537130

[26] Jankovic J. Botulinum toxin in the treatment of dystonic tics. Mov Disord. (1994) 9:347–9. 10.1002/mds.870090315 8041378

[27] Johnson KA Fletcher PT Servello D Bona A Porta M Ostrem JL. Image-based analysis and long-term clinical outcomes of deep brain stimulation for Tourette syndrome: a multisite study. J Neurol Neurosurg Psychiatry. (2019) 90:1078–90. 10.1136/jnnp-2019-320379 31129620 PMC6744301

[28] Martinez-Ramirez D Jimenez-Shahed J Leckman JF Porta M Servello D Meng F-G. Efficacy and safety of deep brain stimulation in tourette syndrome: the international tourette syndrome deep brain stimulation public database and registry. JAMA Neurol. (2018) 75:353–9. 10.1001/jamaneurol.2017.4317 29340590 PMC5885852

[29] O'Reardon JP Cristancho P Peshek AD. Vagus Nerve Stimulation (VNS) and treatment of depression: to the brainstem and beyond. Psychiatry. (2006) 3:54–63. 21103178 PMC2990624

[30] Robertson MM. Tourette syndrome, associated conditions and the complexities of treatment. Brain. (2000) 123 Pt 3:425–62. 10.1093/brain/123.3.425 10686169

[31] Kobayashi M Pascual-Leone A. Transcranial magnetic stimulation in neurology. Lancet Neurol. (2003) 2:145–56. 10.1016/S1474-4422(03)00321-1 12849236

[32] Paulus W. Transcranial electrical stimulation. (tES - tDCS; tRNS, tACS) methods. Neuropsychol Rehabil. (2011) 21:602–17. 10.1080/09602011.2011.557292 21819181

[33] Krishnan C Santos L Peterson MD Ehinger M. Safety of noninvasive brain stimulation in children and adolescents. Brain Stimulation. (2015) 8:76–87. 10.1016/j.brs.2014.10.012 25499471 PMC4459719

[34] Pascual-Leone A Tormos JM Keenan J Tarazona F Cañete C Catalá MD. Study and modulation of human cortical excitability with transcranial magnetic stimulation. J Clin Neurophysiol. (1998) 15:333–43. 10.1097/00004691-199807000-00005 9736467

[35] Paulus W Peterchev AV Ridding M Transcranial electric and magnetic stimulation. In: Handbook of Clinical Neurology. Amsterdam: Elsevier (2013). P. 329–342. 10.1016/B978-0-444-53497-2.00027-9 24112906

[36] Zucker RS Regehr WG. Short-term synaptic plasticity. Annu Rev Physiol. (2002) 64:355–405. 10.1146/annurev.physiol.64.092501.114547 11826273

[37] Shors TJ Matzel LD. Long-term potentiation: what's learning got to do with it? Behav Brain Sci. (1997) 20:597–614. 10.1017/S0140525X97001593 10097007

[38] Lynch MA. Long-term potentiation and memory. Physiol Rev. (2004) 84:87–136. 10.1152/physrev.00014.2003 14715912

[39] Reed T Cohen Kadosh R. Transcranial electrical stimulation. (tES) mechanisms and its effects on cortical excitability and connectivity. J Inherit Metab Dis. (2018) 41:1123–30. 10.1007/s10545-018-0181-4 30006770 PMC6326965

[40] Bikson M Inoue M Akiyama H Deans JK Fox JE Miyakawa H. Effects of uniform extracellular DC electric fields on excitability in rat hippocampal slices *in vitro*: modulation of neuronal function by electric fields. J Physiol. (2004) 557:175–90. 10.1113/jphysiol.2003.055772 14978199 PMC1665051

[41] Stagg CJ Bachtiar V Johansen-Berg H. The role of GABA in human motor learning. Curr Biol. (2011) 21:480–4. 10.1016/j.cub.2011.01.069 21376596 PMC3063350

[42] Hunter MA Coffman BA Gasparovic C Calhoun VD Trumbo MC Clark VP. Baseline effects of transcranial direct current stimulation on glutamatergic neurotransmission and large-scale network connectivity. Brain Res. (2015) 1594:92–107. 10.1016/j.brainres.2014.09.066 25312829 PMC4358793

[43] Huang Y-Z Edwards MJ Rounis E Bhatia KP Rothwell JC. Theta burst stimulation of the human motor cortex. Neuron. (2005) 45:201–6. 10.1016/j.neuron.2004.12.033 15664172

[44] Brownjohn PW Reynolds JNJ Matheson N Fox J Shemmell JBH. The effects of individualized theta burst stimulation on the excitability of the human motor system. Brain Stimul. (2014) 7:260–8. 10.1016/j.brs.2013.12.007 24439960

[45] Moll GH Wischer S Heinrich H Tergau F Paulus W Rothenberger A. Deficient motor control in children with tic disorder: evidence from transcranial magnetic stimulation. Neurosci Letters. (1999) 272:37–40. 10.1016/S0304-3940(99)00575-3 10507537

[46] Gilbert DL Sallee FR Zhang J Lipps TD Wassermann EM. Transcranial magnetic stimulation-evoked cortical inhibition: a consistent marker of attention-deficit/hyperactivity disorder scores in tourette syndrome. Biol Psychiatry. (2005) 57:1597–600. 10.1016/j.biopsych.2005.02.022 15953499

[47] Orth M Amann B Robertson MM Rothwell JC. Excitability of motor cortex inhibitory circuits in Tourette syndrome before and after single dose nicotine. Brain. (2005) 128:1292–300. 10.1093/brain/awh473 15774505

[48] Heise K-F Steven B Liuzzi G Thomalla G Jonas M Muller-Vahl K. Altered modulation of intracortical excitability during movement preparation in Gilles de la Tourette syndrome. Brain. (2010) 133:580–90. 10.1093/brain/awp299 20008030

[49] Brandt VC Niessen E Ganos C Kahl U Bäumer T Münchau A. Altered synaptic plasticity in tourette's syndrome and its relationship to motor skill learning. PLoS ONE. (2014) 9:e98417. 10.1371/journal.pone.0098417 24878665 PMC4039486

[50] Tübing J Gigla B Brandt VC Verrel J Weissbach A Beste C. Associative plasticity in supplementary motor area - motor cortex pathways in Tourette syndrome. Sci Rep. (2018) 8:11984. 10.1038/s41598-018-30504-8 30097615 PMC6086903

[51] Orth M Münchau A Rothwell JC. Corticospinal system excitability at rest is associated with tic severity in tourette syndrome. Biol Psychiatry. (2008) 64:248–51. 10.1016/j.biopsych.2007.12.009 18243162

[52] Pépés SE Draper A Jackson GM Jackson SR. Effects of age on motor excitability measures from children and adolescents with Tourette syndrome. Dev Cogn Neurosci. (2016) 19:78–86. 10.1016/j.dcn.2016.02.005 26934638 PMC6988104

[53] Draper A Jude L Jackson GM Jackson SR. Motor excitability during movement preparation in Tourette syndrome. J Neuropsychol. (2015) 9:33–44. 10.1111/jnp.12033 24283505 PMC4374703

[54] Draper A Stephenson MC Jackson GM Pépés S Morgan PS Morris PG. Increased GABA contributes to enhanced control over motor excitability in tourette syndrome. Curr Biol. (2014) 24:2343–7. 10.1016/j.cub.2014.08.038 25264251 PMC4188813

[55] Ganos C Rocchi L Latorre A Hockey L Palmer C Joyce EM. Motor cortical excitability during voluntary inhibition of involuntary tic movements: the motor neurophysiology of tic inhibition. Mov Disord. (2018) 33:1804–9. 10.1002/mds.27479 30379360

[56] Cantello R Gianelli M Civardi C Mutani R. Magnetic brain stimulation: the silent period after the motor evoked potential. Neurology. (1992) 42:1951–9. 10.1212/WNL.42.10.1951 1407578

[57] Inghilleri M Berardelli A Cruccu G Manfredi M. Silent period evoked by transcranial stimulation of the human cortex and cervicomedullary junction. J Physiol. (1993) 466:521–34. 8410704 PMC1175490

[58] Wolters A Ziemann U Benecke R The Cortical Silent Period. Epstein CM Wassermann EM Ziemann U editors. Oxford: Oxford University Press (2012). 10.1093/oxfordhb/9780198568926.013.0010

[59] Triggs WJ Macdonell RA Cros D Chiappa KH Shahani BT Day BJ. Motor inhibition and excitation are independent effects of magnetic cortical stimulation. Ann Neurol. (1992) 32:345–51. 10.1002/ana.410320307 1416804

[60] Orth M Rothwell JC. The cortical silent period: intrinsic variability and relation to the waveform of the transcranial magnetic stimulation pulse. Clin Neurophysiol. (2004) 115:1076–82. 10.1016/j.clinph.2003.12.025 15066533

[61] Chen R Lozano AM Ashby P. Mechanism of the silent period following transcranial magnetic stimulation. Evidence from epidural recordings. Exp Brain Res. (1999) 128:539–42. 10.1007/s002210050878 10541749

[62] Poston B Kukke SN Paine RW Francis S Hallett M. Cortical silent period duration and its implications for surround inhibition of a hand muscle. Eur J Neurosci. (2012) 36:2964–71. 10.1111/j.1460-9568.2012.08212.x 22775302 PMC3463678

[63] Gilbert DL Bansal AS Sethuraman G Sallee FR Zhang J Lipps T. Association of cortical disinhibition with tic, ADHD, and OCD severity in Tourette syndrome. Mov Disord. (2004) 19:416–25. 10.1002/mds.20044 15077239

[64] Ziemann U Paulus W Rothenberger A. Decreased motor inhibition in Tourette's disorder: evidence from transcranial magnetic stimulation. AJP. (1997) 154:1277–84. 10.1176/ajp.154.9.1277 9286189

[65] Orth M. Transcranial magnetic stimulation in Gilles de la Tourette syndrome. J Psychosom Res. (2009) 67:591–8. 10.1016/j.jpsychores.2009.07.014 19913663

[66] Di Lazzaro V Pilato F Dileone M Ranieri F Ricci V Profice P. GABAA receptor subtype specific enhancement of inhibition in human motor cortex. J Physiol. (2006) 575:721–6. 10.1113/jphysiol.2006.114694 16809358 PMC1995685

[67] Tokimura H Di Lazzaro V Tokimura Y Oliviero A Profice P Insola A. Short latency inhibition of human hand motor cortex by somatosensory input from the hand. J Physiol. (2000) 523:503–13. 10.1111/j.1469-7793.2000.t01-1-00503.x 10699092 PMC2269813

[68] Porter R Lemon R Corticospinal Function and Voluntary Movement. Oxford: Oxford University Press (1995)

[69] Bertolasi L Priori A Tinazzi M Bertasi V Rothwell JC. Inhibitory action of forearm flexor muscle afferents on corticospinal outputs to antagonist muscles in humans. J Physiol. (1998) 511:947–56. 10.1111/j.1469-7793.1998.947bg.x 9714872 PMC2231145

[70] Orth M Rothwell JC. Motor cortex excitability and comorbidity in Gilles de la Tourette syndrome. J Neurol Neurosurg Psychiatry. (2009) 80:29–34. 10.1136/jnnp.2008.149484 18931001

[71] Munchau A Bloem BR Thilo KV Trimble MR Rothwell JC Robertson MM. Repetitive transcranial magnetic stimulation for Tourette syndrome. Neurology. (2002) 59:1789–91. 10.1212/01.WNL.0000036615.25044.50 12473773

[72] Orth M Kirby R Richardson MP Snijders AH Rothwell JC Trimble MR. Subthreshold rTMS over pre-motor cortex has no effect on tics in patients with Gilles de la Tourette syndrome. Clin Neurophysiol. (2005) 116:764–8. 10.1016/j.clinph.2004.10.003 15792884

[73] Georgiou N Bradshaw JL Phillips JG Bradshaw JA Chiu E. The Simon effect and attention deficits in Gilles de la Tourette's syndrome and Huntington's disease. Brain. (1995) 118:1305–18. 10.1093/brain/118.5.1305 7496788

[74] Dursun SM Burke JG Reveley MA. Antisaccade eye movement abnormalities in Tourette syndrome: evidence for cortico-striatal network dysfunction? J Psychopharmacol. (2000) 14:37–9. 10.1177/026988110001400104 10757251

[75] Crawford S Channon S Robertson MM. Tourette's syndrome: performance on tests of behavioural inhibition, working memory and gambling. J Child Psychol Psychiat. (2005) 46:1327–36. 10.1111/j.1469-7610.2005.01419.x 16313433

[76] Mantovani A Lisanby SH Pieraccini F Ulivelli M Castrogiovanni P Rossi S. Repetitive transcranial magnetic stimulation (rTMS) in the treatment of obsessive–compulsive disorder (OCD) and Tourette's syndrome (TS). Int J Neuropsychopharm. (2005) 9:95. 10.1017/S1461145705005729 15982444

[77] Channon S Drury H Martinos M Robertson MM Orth M Crawford S. Tourette's syndrome (TS): inhibitory performance in adults with uncomplicated TS. Neuropsychology. (2009) 23:359–66. 10.1037/a0014552 19413449

[78] Kwon HJ Lim WS Lim MH Lee SJ Hyun JK Chae J-H. 1-Hz low frequency repetitive transcranial magnetic stimulation in children with Tourette's syndrome. Neurosci Letters. (2011) 492:1–4. 10.1016/j.neulet.2011.01.007 21256925

[79] Le K Liu L Sun M Hu L Xiao N. Transcranial magnetic stimulation at 1 Hertz improves clinical symptoms in children with Tourette syndrome for at least 6 months. J Clin Neurosci. (2013) 20:257–62. 10.1016/j.jocn.2012.01.049 23238046

[80] Wylie S Claassen D Kanoff K Ridderinkhof K van den Wildenberg W. Impaired inhibition of prepotent motor actions in patients with Tourette syndrome. J Psychiatry Neurosci. (2013) 38:349–56. 10.1503/jpn.120138 23820185 PMC3756119

[81] Serrien DJ. Motor inhibition in patients with Gilles de la Tourette syndrome: functional activation patterns as revealed by EEG coherence. Brain. (2004) 128:116–25. 10.1093/brain/awh318 15496435

[82] Roessner V Albrecht B Dechent P Baudewig J Rothenberger A. Normal response inhibition in boys with Tourette syndrome. Behav Brain Funct. (2008) 4:29. 10.1186/1744-9081-4-29 18638368 PMC2491645

[83] Ganos C Kahl U Brandt V Schunke O Bäumer T Thomalla G. The neural correlates of tic inhibition in Gilles de la Tourette syndrome. Neuropsychologia. (2014) 65:297–301. 10.1016/j.neuropsychologia.2014.08.007 25128587

[84] Petruo V Bodmer B Bluschke A Münchau A Roessner V Beste C. Comprehensive Behavioral Intervention for Tics reduces perception-action binding during inhibitory control in Gilles de la Tourette syndrome. Sci Rep. (2020) 10:1174. 10.1038/s41598-020-58269-z 31980733 PMC6981113

[85] Mueller SC Jackson GM Dhalla R Datsopoulos S Hollis CP. Enhanced cognitive control in young people with tourette's syndrome. Curr Biol. (2006) 16:570–3. 10.1016/j.cub.2006.01.064 16546080

[86] Jackson GM Mueller SC Hambleton K Hollis CP. Enhanced cognitive control in Tourette Syndrome during task uncertainty. Exp Brain Res. (2007) 182:357–64. 10.1007/s00221-007-0999-8 17569034

[87] Brandt VC Stock A-K Münchau A Beste C. Evidence for enhanced multi-component behaviour in Tourette syndrome – an EEG study. Sci Rep. (2017) 7:7722. 10.1038/s41598-017-08158-9 28798371 PMC5552788

[88] Brandt VC Beck C Sajin V Baaske MK Bäumer T Beste C. Temporal relationship between premonitory urges and tics in Gilles de la Tourette syndrome. Cortex. (2016) 77:24–37. 10.1016/j.cortex.2016.01.008 26922503

[89] Morand-Beaulieu S Grot S Lavoie J Leclerc JB Luck D Lavoie ME. The puzzling question of inhibitory control in Tourette syndrome: a meta-analysis. Neurosci Biobehav Rev. (2017) 80:240–62. 10.1016/j.neubiorev.2017.05.006 28502600

[90] Jahanshahi M Obeso I Rothwell JC Obeso JA. A fronto–striato–subthalamic–pallidal network for goal-directed and habitual inhibition. Nat Rev Neurosci. (2015) 16:719–32. 10.1038/nrn4038 26530468

[91] Jahanshahi M Rothwell JC. Inhibitory dysfunction contributes to some of the motor and non-motor symptoms of movement disorders and psychiatric disorders. Phil Trans R Soc B. (2017) 372:20160198. 10.1098/rstb.2016.0198 28242732 PMC5332857

[92] Aron AR. From reactive to proactive and selective control: developing a richer model for stopping inappropriate responses. Biol Psychiatry. (2011) 69:e55–68. 10.1016/j.biopsych.2010.07.024 20932513 PMC3039712

[93] Rawji V Modi S Latorre A Rocchi L Hockey L Bhatia K. Impaired automatic but intact volitional inhibition in primary tic disorders. Brain. (2020) 143:906–19. 10.1093/brain/awaa024 32125364 PMC7089661

[94] Stenner M-P Baumgaertel C Heinze H-J Ganos C Müller-Vahl KR. Intact automatic motor inhibition in patients with tourette syndrome. Mov Disord. (2018) 33:1800–4. 10.1002/mds.27493 30485912

[95] Sumner P Nachev P Morris P Peters AM Jackson SR Kennard C. Human medial frontal cortex mediates unconscious inhibition of voluntary action. Neuron. (2007) 54:697–711. 10.1016/j.neuron.2007.05.016 17553420 PMC1890004

[96] D'Ostilio K Collette F Phillips C Garraux G. Evidence for a role of a cortico-subcortical network for automatic and unconscious motor inhibition of manual responses. PLoS ONE. (2012) 7:e48007. 10.1371/journal.pone.0048007 23110158 PMC3480469

[97] Bestmann S Baudewig J Siebner HR Rothwell JC Frahm J. Functional MRI of the immediate impact of transcranial magnetic stimulation on cortical and subcortical motor circuits. Eur J Neurosci. (2004) 19:1950–62. 10.1111/j.1460-9568.2004.03277.x 15078569

[98] Amassian VE Cracco RQ Maccabee PJ Cracco JB Rudell AP Eberle L. Transcranial magnetic stimulation in study of the visual pathway. J Clin Neurophysiol. (1998) 15:288–304. 10.1097/00004691-199807000-00002 9736464

[99] Lefaucheur J-P. Transcranial magnetic stimulation. Handb Clin Neurol. (2019) 160:559–80. 10.1016/B978-0-444-64032-1.00037-0 31277876

[100] Sparing R Buelte D Meister IG Paus T Fink GR. Transcranial magnetic stimulation and the challenge of coil placement: a comparison of conventional and stereotaxic neuronavigational strategies. Hum Brain Mapp. (2008) 29:82–96. 10.1002/hbm.20360 17318831 PMC6871049

[101] Herwig U Schönfeldt-Lecuona C Wunderlich AP von Tiesenhausen C Thielscher A Walter H. The navigation of transcranial magnetic stimulation. Psychiatry Res. (2001) 108:123–31. 10.1016/S0925-4927(01)00121-4 11738546

[102] Landeros-Weisenberger A Mantovani A Motlagh MG de Alvarenga PG Katsovich L Leckman JF. Randomized sham controlled double-blind trial of repetitive transcranial magnetic stimulation for adults with severe tourette syndrome. Brain Stimulation. (2015) 8:574–81. 10.1016/j.brs.2014.11.015 25912296 PMC4454615

[103] Mrakic-Sposta S Marceglia S Mameli F Dilena R Tadini L Priori A. Transcranial direct current stimulation in two patients with Tourette syndrome. Mov Disord. (2008) 23:2259–61. 10.1002/mds.22292 18785641

[104] Carvalho S Gonçalves ÓF Soares JM Sampaio A Macedo F Fregni F. Sustained effects of a neural-based intervention in a refractory case of tourette syndrome. Brain Stimulation. (2015) 8:657–9. 10.1016/j.brs.2014.12.008 25616936

[105] Eapen V Baker R Walter A Raghupathy V Wehrman JJ Sowman PF. The role of transcranial direct current stimulation (tDCS) in tourette syndrome: a review and preliminary findings. Brain Sci. (2017) 7:161. 10.3390/brainsci7120161 29292730 PMC5742764

[106] Dyke K Jackson GM Nixon E Jackson SR. Effects of single-session cathodal transcranial direct current stimulation on tic symptoms in Tourette's syndrome. Exp Brain Res. (2019) 237:2853–63. 10.1007/s00221-019-05637-5 31463531 PMC6794240

[107] Wu SW Maloney T Gilbert DL Dixon SG Horn PS Huddleston DA. Functional MRI-navigated repetitive transcranial magnetic stimulation over supplementary motor area in chronic tic disorders. Brain Stimul. (2014) 7:212–8. 10.1016/j.brs.2013.10.005 24268723

[108] Eidelberg D Moeller JR Antonini A Kazumata K Dhawan V Budman C. The metabolic anatomy of Tourette's syndrome. Neurology. (1997) 48:927–33. 10.1212/WNL.48.4.927 9109879

[109] Peterson BS Skudlarski P Anderson AW Zhang H Gatenby JC Lacadie CM. A functional magnetic resonance imaging study of tic suppression in Tourette syndrome. Arch Gen Psychiatry. (1998) 55:326–33. 10.1001/archpsyc.55.4.326 9554428

[110] Stern E Silbersweig DA Chee KY Holmes A Robertson MM Trimble M. A functional neuroanatomy of tics in Tourette syndrome. Arch Gen Psychiatry. (2000) 57:741–8. 10.1001/archpsyc.57.8.741 10920461

[111] Akkal D Dum RP Strick PL. Supplementary motor area and presupplementary motor area: targets of basal ganglia and cerebellar output. J Neurosci. (2007) 27:10659–73. 10.1523/JNEUROSCI.3134-07.2007 17913900 PMC6672811

[112] Nachev P Kennard C Husain M. Functional role of the supplementary and pre-supplementary motor areas. Nat Rev Neurosci. (2008) 9:856–69. 10.1038/nrn2478 18843271

[113] Habas C. Functional connectivity of the human rostral and caudal cingulate motor areas in the brain resting state at 3T. Neuroradiology. (2010) 52:47–59. 10.1007/s00234-009-0572-1 19629462

[114] Zhang S Ide JS Li CR. Resting-state functional connectivity of the medial superior frontal cortex. Cerebral Cortex. (2012) 22:99–111. 10.1093/cercor/bhr088 21572088 PMC3236794

[115] Hampson M Tokoglu F King RA Constable RT Leckman JF. Brain areas coactivating with motor cortex during chronic motor tics and intentional movements. Biol Psychiatry. (2009) 65:594–9. 10.1016/j.biopsych.2008.11.012 19111281 PMC2679868

[116] Bohlhalter S. Neural correlates of tic generation in Tourette syndrome: an event-related functional MRI study. Brain. (2006) 129:2029–37. 10.1093/brain/awl050 16520330

[117] Wang Z Maia TV Marsh R Colibazzi T Gerber A Peterson BS. The neural circuits that generate tics in Tourette's syndrome. Am J Psychiatry. (2011) 168:1326–37. 10.1176/appi.ajp.2011.09111692 21955933 PMC4246702

[118] Neuner I Werner CJ Arrubla J Stöcker T Ehlen C Wegener HP. Imaging the where and when of tic generation and resting state networks in adult Tourette patients. Front Hum Neurosci. (2014) 8:362. 10.3389/fnhum.2014.00362 24904391 PMC4035756

[119] Hampson M Scheinost D Qiu M Bhawnani J Lacadie CM Leckman JF. Biofeedback of real-time functional magnetic resonance imaging data from the supplementary motor area reduces functional connectivity to subcortical regions. Brain Connectivity. (2011) 1:91–8. 10.1089/brain.2011.0002 22432958 PMC3621512

[120] Sukhodolsky DG Walsh C Koller WN Eilbott J Rance M Fulbright RK. Randomized, sham-controlled trial of real-time functional magnetic resonance imaging neurofeedback for tics in adolescents with tourette syndrome. Biol Psychiatry. (2019) 87:1063–70. 10.1016/j.biopsych.2019.07.035 31668476 PMC7015800

[121] Thomalla G Jonas M Bäumer T Siebner HR Biermann-Ruben K Ganos C. Costs of control: decreased motor cortex engagement during a Go/NoGo task in Tourette's syndrome. Brain. (2014) 137:122–36. 10.1093/brain/awt288 24176975

[122] Swick D Ashley V Turken U. Are the neural correlates of stopping and not going identical? Quantitative meta-analysis of two response inhibition tasks. NeuroImage. (2011) 56:1655–65. 10.1016/j.neuroimage.2011.02.070 21376819

[123] Rae CL Hughes LE Weaver C Anderson MC Rowe JB. Selection and stopping in voluntary action: a meta-analysis and combined fMRI study. NeuroImage. (2014) 86:381–91. 10.1016/j.neuroimage.2013.10.012 24128740 PMC3898966

[124] Bari A Robbins TW. Inhibition and impulsivity: behavioral and neural basis of response control. Prog Neurobiol. (2013) 108:44–79. 10.1016/j.pneurobio.2013.06.005 23856628

[125] Mückschel M Stock A-K Dippel G Chmielewski W Beste C. Interacting sources of interference during sensorimotor integration processes. Neuroimage. (2016) 125:342–9. 10.1016/j.neuroimage.2015.09.075 26596550

[126] Mückschel M Dippel G Beste C. Distinguishing stimulus and response codes in theta oscillations in prefrontal areas during inhibitory control of automated responses. Hum Brain Mapp. (2017) 38:5681–90. 10.1002/hbm.23757 28782869 PMC6867003

[127] Bensmann W Zink N Werner A Beste C Stock A-K Acute alcohol effects on response inhibition depend on response automatization, but not on GABA or glutamate levels in the ACC and striatum. J Clin Med. (2020) 9:481 10.3390/jcm9020481 PMC7073826 32050509

[128] Franzkowiak S Pollok B Biermann-Ruben K Südmeyer M Paszek J Thomalla G. Motor-cortical interaction in gilles de la tourette syndrome. PLoS ONE. (2012) 7:e27850. 10.1371/journal.pone.0027850 22238571 PMC3251574

[129] Cunnington R Windischberger C Deecke L Moser E. The preparation and execution of self-initiated and externally-triggered movement: a study of event-related fMRI. Neuroimage. (2002) 15:373–85. 10.1006/nimg.2001.0976 11798272

[130] Ganos C. Tics and Tourette's: update on pathophysiology and tic control. Curr Opin Neurol. (2016) 29:513–8. 10.1097/WCO.0000000000000356 27310537

[131] Kawohl W Brühl A Krowatschek G Ketteler D Herwig U. Functional magnetic resonance imaging of tics and tic suppression in Gilles de la Tourette syndrome. World J Biol Psychiatry. (2009) 10:567–70. 10.1080/15622970802118356 18609432

[132] van der Salm SMA van der Meer JN Cath DC Groot PFC van der Werf YD Brouwers E. Distinctive tics suppression network in Gilles de la Tourette syndrome distinguished from suppression of natural urges using multimodal imaging. Neuroimage Clin. (2018) 20:783–92. 10.1016/j.nicl.2018.09.014 30268027 PMC6169325

[133] Aron AR Robbins TW Poldrack RA. Inhibition and the right inferior frontal cortex: one decade on. Trends Cogn Sci. (2014) 18:177–85. 10.1016/j.tics.2013.12.003 24440116

[134] Dippel G Beste C. A causal role of the right inferior frontal cortex in implementing strategies for multi-component behaviour. Nat Commun. (2015) 6:6587. 10.1038/ncomms7587 25850926

[135] Tinaz S Malone P Hallett M Horovitz SG. Role of the right dorsal anterior insula in the urge to tic in tourette syndrome: right dorsal anterior insula in tourette. Mov Disord. (2015) 30:1190–7. 10.1002/mds.26230 25855089 PMC5088605

[136] Cohen AJ Leckman JF. Sensory phenomena associated with Gilles de la Tourette's syndrome. J Clin Psychiatry. (1992) 53:319–23. 1517194

[137] Belluscio BA Jin L Watters V Lee TH Hallett M. Sensory sensitivity to external stimuli in Tourette syndrome patients. Mov Disord. (2011) 26:2538–43. 10.1002/mds.23977 22038938 PMC3240739

[138] Buse J Beste C Herrmann E Roessner V. Neural correlates of altered sensorimotor gating in boys with Tourette Syndrome: a combined EMG/fMRI study. World J Biol Psychiatry. (2016) 17:187–97. 10.3109/15622975.2015.1112033 26624257

[139] Sowell ER Kan E Yoshii J Thompson PM Bansal R Xu D. Thinning of sensorimotor cortices in children with Tourette syndrome. Nat Neurosci. (2008) 11:637–9. 10.1038/nn.2121 18488025 PMC2605107

[140] Thomalla G Siebner HR Jonas M Bäumer T Biermann-Ruben K Hummel F. Structural changes in the somatosensory system correlate with tic severity in Gilles de la Tourette syndrome. Brain. (2009) 132:765–77. 10.1093/brain/awn339 19136548

[141] Church J. Task control signals in pediatric Tourette syndrome show evidence of immature and anomalous functional activity. Front Hum Neurosci. (2009) 3:38. 10.3389/neuro.09.038.2009 19949483 PMC2784679

[142] Worbe Y Malherbe C Hartmann A Pélégrini-Issac M Messé A Vidailhet M. Functional immaturity of cortico-basal ganglia networks in Gilles de la Tourette syndrome. Brain. (2012) 135:1937–46. 10.1093/brain/aws056 22434213

[143] Eickhoff SB Jbabdi S Caspers S Laird AR Fox PT Zilles K. Anatomical and functional connectivity of cytoarchitectonic areas within the human parietal operculum. J Neurosci. (2010) 30:6409–21. 10.1523/JNEUROSCI.5664-09.2010 20445067 PMC4791040

[144] Geng JJ Vossel S. Re-evaluating the role of TPJ in attentional control: contextual updating? Neurosci Biobehav Rev. (2013) 37:2608–20. 10.1016/j.neubiorev.2013.08.010 23999082 PMC3878596

[145] Schunke O Grashorn W Kahl U Schöttle D Haggard P Münchau A. Quantitative sensory testing in adults with tourette syndrome. Parkinsonism Related Disorders. (2016) 24:132–6. 10.1016/j.parkreldis.2016.01.006 26818628

[146] Münchau A Thomalla G Roessner V. Somatosensory phenomena and the role of sensorimotor circuits in Gilles de la Tourette syndrome. Z Kinder Jugendpsychiatr Psychother. (2011) 39:161–7. 10.1024/1422-4917/a000091 21563107

[147] Orth M Münchau A. Transcranial magnetic stimulation studies of sensorimotor networks in tourette syndrome. Behav Neurol. (2013) 27:57–64. 10.1155/2013/349137 23187144 PMC5214886

[148] Cheng B Braass H Ganos C Treszl A Biermann-Ruben K Hummel FC. Altered intrahemispheric structural connectivity in Gilles de la Tourette syndrome. NeuroImage Clin. (2014) 4:174–81. 10.1016/j.nicl.2013.11.011 24371800 PMC3872720

[149] Serrien DJ Nirkko AC Loher TJ Lövblad K-O Burgunder J-M Wiesendanger M. Movement control of manipulative tasks in patients with Gilles de la Tourette syndrome. Brain. (2002) 125:290–300. 10.1093/brain/awf024 11844729

[150] Nowak DA Rothwell J Topka H Robertson MM Orth M. Grip force behavior in Gilles de la Tourette syndrome. Mov Disord. (2005) 20:217–23. 10.1002/mds.20309 15382208

[151] Dippel G Chmielewski W Mückschel M Beste C. Response mode-dependent differences in neurofunctional networks during response inhibition: an EEG-beamforming study. Brain Struct Funct. (2016) 221:4091–101. 10.1007/s00429-015-1148-y 26608829

[152] Friedrich J Beste C. The impact of stimulus modality on the processing of conflicting sensory information during response inhibition. Neuroscience. (2019) 410:191–201. 10.1016/j.neuroscience.2019.05.010 31100340

[153] Opitz A Beste C Stock AK. Using temporal EEG signal decomposition to identify specific neurophysiological correlates of distractor-response bindings proposed by the theory of event coding. Neuroimage. (2020) 209:116524. 10.1016/j.neuroimage.2020.116524 31926281

[154] Hommel B Müsseler J Aschersleben G Prinz W. The theory of event coding (TEC): a framework for perception and action planning. Behav Brain Sci. (2001) 24:849–78. 10.1017/S0140525X01000103 12239891

[155] Colzato LS Warrens MJ Hommel B. Priming and binding in and across perception and action: a correlational analysis of the internal structure of event files. Q J Exp Psychol. (2006) 59:1785–804. 10.1080/17470210500438304 16945860

[156] Stefan K Kunesch E Cohen LG Benecke R Classen J. Induction of plasticity in the human motor cortex by paired associative stimulation. Brain. (2000) 123:572–84. 10.1093/brain/123.3.572 10686179

[157] Ridding MC Ziemann U. Determinants of the induction of cortical plasticity by non-invasive brain stimulation in healthy subjects: induction of cortical plasticity by non-invasive brain stimulation. J Physiol. (2010) 588:2291–304. 10.1113/jphysiol.2010.190314 20478978 PMC2915507

[158] Suppa A Marsili L Di Stasio F Berardelli I Roselli V Pasquini M. Cortical and brainstem plasticity in Tourette syndrome and obsessive-compulsive disorder: M1 and Brainstem LTP/LTD-Like Plasticity In GTS and OCD. Mov Disord. (2014) 29:1523–31. 10.1002/mds.25960 24996148

[159] Suppa A Belvisi D Bologna M Marsili L Berardelli I Moretti G. Abnormal cortical and brain stem plasticity in Gilles de la Tourette syndrome. Mov Disord. (2011) 26:1703–10. 10.1002/mds.23706 21442662

[160] Martín-Rodríguez JF Ruiz-Rodríguez MA Palomar FJ Cáceres-Redondo MT Vargas L Porcacchia P. Aberrant cortical associative plasticity associated with severe adult Tourette syndrome. Mov Disord. (2015) 30:431–5. 10.1002/mds.26151 25649686

[161] Goldsworthy MR Müller-Dahlhaus F Ridding MC Ziemann U. Inter-subject Variability of LTD-like plasticity in human motor cortex: a matter of preceding motor activation. Brain Stimulation. (2014) 7:864–70. 10.1016/j.brs.2014.08.004 25216649

[162] Do M Kirkovski M Davies CB Bekkali S Byrne LK Enticott PG. Intra- and inter-regional priming of ipsilateral human primary motor cortex with continuous theta burst stimulation does not induce consistent neuroplastic effects. Front Hum Neurosci. (2018) 12:123. 10.3389/fnhum.2018.00123 29651241 PMC5884878

[163] Yang C Guo Z Peng H Xing G Chen H McClure MA. Repetitive transcranial magnetic stimulation therapy for motor recovery in Parkinson's disease: a meta-analysis. Brain Behav. (2018) 8:e01132. 10.1002/brb3.1132 30264518 PMC6236247

[164] Marsili L Suppa A Di Stasio F Belvisi D Upadhyay N Berardelli I. BDNF and LTP-/LTD-like plasticity of the primary motor cortex in Gilles de la Tourette syndrome. Exp Brain Res. (2017) 235:841–50. 10.1007/s00221-016-4847-6 27900437

[165] Mehrabi N McPhee J Model-based control of biomechatronic systems. In Handbook of Biomechatronics. Elsevier (2019). p. 95–126. 10.1016/B978-0-12-812539-7.00004-0

[166] Nathan DA Center S Wu C Keller W. An implantable synchronous pacemaker for the long term correction of complete heart block. Am J Cardiol. (1963) 11:362–7. 10.1016/0002-9149(63)90130-9 13937691

[167] Deuschl G Schade-Brittinger C Krack P Volkmann J Schäfer H Bötzel K. A randomized trial of deep-brain stimulation for Parkinson's disease. N Engl J Med. (2006) 355:896–908. 10.1056/NEJMoa060281 16943402

[168] Vidailhet M Vercueil L Houeto J-L Krystkowiak P Lagrange C Yelnik J. Bilateral, pallidal, deep-brain stimulation in primary generalised dystonia: a prospective 3 year follow-up study. Lancet Neurol. (2007) 6:223–9. 10.1016/S1474-4422(07)70035-2 17303528

[169] Einevoll GT Kayser C Logothetis NK Panzeri S. Modelling and analysis of local field potentials for studying the function of cortical circuits. Nat Rev Neurosci. (2013) 14:770–85. 10.1038/nrn3599 24135696

[170] Hebb AO Zhang JJ Mahoor MH Tsiokos C Matlack C Chizeck HJ. Creating the feedback loop. Neurosurg Clinics North Am. (2014) 25:187–204. 10.1016/j.nec.2013.08.006 24262909 PMC4058859

[171] Little S Pogosyan A Neal S Zavala B Zrinzo L Hariz M. Adaptive deep brain stimulation in advanced Parkinson disease: adaptive DBS in PD. Ann Neurol..(2013) 74:449–57. 10.1002/ana.23951 23852650 PMC3886292

[172] Cagle JN Okun MS Opri E Cernera S Molina R Foote KD. Differentiating tic electrophysiology from voluntary movement in the human thalamocortical circuit. J Neurol Neurosurg Psychiatry. (2020) 91:533–9. 10.1136/jnnp-2019-321973 32139653 PMC7296862

[173] Hallett M. Transcranial magnetic stimulation and the human brain. Nature. (2000) 406:147–50. 10.1038/35018000 10910346

[174] Burle B Spieser L Roger C Casini L Hasbroucq T Vidal F. Spatial and temporal resolutions of EEG: is it really black and white? A scalp current density view. Int J Psychophysiol. (2015) 97:210–20. 10.1016/j.ijpsycho.2015.05.004 25979156 PMC4548479

[175] Mutanen T. TMS-evoked changes in brain-state dynamics quantified by using EEG data. Front Hum Neurosci. (2013) 7:155. 10.3389/fnhum.2013.00155 23630486 PMC3635036

[176] Zrenner C Desideri D Belardinelli P Ziemann U. Real-time EEG-defined excitability states determine efficacy of TMS-induced plasticity in human motor cortex. Brain Stimulation. (2018) 11:374–89. 10.1016/j.brs.2017.11.016 29191438

[177] Robertson MM. The Gilles de la Tourette syndrome: the current status. Br J Psychiatry. (1989) 154:147–69. 10.1192/bjp.154.2.147 2673473

[178] Bernabei M Andreoni G Mendez Garcia MO Piccini L Aletti F Sassi M Automatic detection of tic activity in the Tourette Syndrome. :In 2010 Annual International Conference of the IEEE Engineering in Medicine and Biology. Buenos Aires: IEEE.(2010). p. 422–425. 10.1109/IEMBS.2010.5627374 21096762

[179] Brandt VC Hermanns J Beck C Bäumer T Zurowski B Münchau A. The temporal relationship between premonitory urges and covert compulsions in patients with obsessive-compulsive disorder. Psychiatry Res. (2018) 262:6–12. 10.1016/j.psychres.2018.01.041 29407570

[180] Dieringer M Beck C Verrel J Münchau A Zurowski B Brandt V. Quality and temporal properties of premonitory urges in patients with skin picking disorder. Cortex. (2019) 121:125–34. 10.1016/j.cortex.2019.08.015 31605885

[181] Sheehan DV Lecrubier Y Sheehan KH Amorim P Janavs J Weiller E. The Mini-International Neuropsychiatric Interview (M.I.N.I.): the development and validation of a structured diagnostic psychiatric interview for DSM-IV and ICD-10. J Clin Psychiatry. (1998) 59(Suppl. 20), 22–33. 9881538

[182] Harrison AG Nay S Armstrong IT. Diagnostic accuracy of the conners' adult ADHD rating scale in a postsecondary population. J Atten Disord. (2019) 23:1829–37. 10.1177/1087054715625299 26794674

[183] Goodman WK Price LH Rasmussen SA Mazure C Fleischmann RL Hill CL. The yale-brown obsessive compulsive scale. I Development, use, and reliability. Arch Gen Psychiatry. (1989) 46:1006–11. 10.1001/archpsyc.1989.01810110048007 2684084

[184] Goetz CG Pappert EJ Louis ED Raman R Leurgans S. Advantages of a modified scoring method for the rush video-based tic rating scale. Mov Disord. (1999) 14:502–6. 10.1002/1531-825719990514:3<502::AID-MDS1020>3.0.CO;2-G 10348478

[185] Leckman JF Riddle MA Hardin MT Ort SI Swartz KL Stevenson J. The Yale Global Tic Severity Scale: initial testing of a clinician-rated scale of tic severity. J Am Acad Child Adolesc Psychiatry. (1989) 28:566–73. 10.1097/00004583-198907000-00015 2768151

[186] Woods DW Piacentini J Himle MB Chang S. Premonitory Urge for Tics Scale. (PUTS): initial psychometric results and examination of the premonitory urge phenomenon in youths with Tic disorders. J Dev Behav Pediatr. (2005) 26:397–403. 10.1097/00004703-200512000-00001 16344654

[187] Rush S Driscoll DA. Current distribution in the brain from surface electrodes. Anesth Analg. (1968) 47:717–23. 10.1213/00000539-196811000-00016 4972743

[188] Dymond AM Coger RW Serafetinides EA. Intracerebral current levels in man during electrosleep therapy. Biol Psychiatry. (1975) 10:101–4. 1120172

[189] Bindman LJ Lippold OCJ Redfearn JWT. The action of brief polarizing currents on the cerebral cortex of the rat (1) during current flow and (2) in the production of long-lasting after-effects. J Physiol. (1964) 172:369–82. 10.1113/jphysiol.1964.sp007425 14199369 PMC1368854

[190] Priori A Berardelli A Rona S Accornero N Manfredi M. Polarization of the human motor cortex through the scalp. Neuroreport. (1998) 9:2257–60. 10.1097/00001756-199807130-00020 9694210

[191] Nitsche MA Paulus W. Excitability changes induced in the human motor cortex by weak transcranial direct current stimulation. J Physiol. (2000) 527:633–9. 10.1111/j.1469-7793.2000.t01-1-00633.x 10990547 PMC2270099

[192] Nitsche MA Paulus W. Sustained excitability elevations induced by transcranial DC motor cortex stimulation in humans. Neurology. (2001) 57:1899–901. 10.1212/WNL.57.10.1899 11723286

[193] Nitsche MA Nitsche MS Klein CC Tergau F Rothwell JC Paulus W. Level of action of cathodal DC polarisation induced inhibition of the human motor cortex. Clin Neurophysiol. (2003) 114:600–4. 10.1016/S1388-2457(02)00412-1 12686268

[194] Priori A. Brain polarization in humans: a reappraisal of an old tool for prolonged non-invasive modulation of brain excitability. Clin Neurophysiol. (2003) 114:589–95. 10.1016/S1388-2457(02)00437-6 12686266

[195] Nitsche MA Cohen LG Wassermann EM Priori A Lang N Antal A. Transcranial direct current stimulation: state of the art 2008. Brain Stimulation. (2008) 1:206–23. 10.1016/j.brs.2008.06.004 20633386

[196] Iyer MB Mattu U Grafman J Lomarev M Sato S Wassermann EM. Safety and cognitive effect of frontal DC brain polarization in healthy individuals. Neurology. (2005) 64:872–5. 10.1212/01.WNL.0000152986.07469.E9 15753425

[197] Jiang J Truong DQ Bikson M Abstract #115: what is theoretically more focal: HD-tDCS or TMS? Brain Stimulation. (2019) 12:e39–e40. 10.1016/j.brs.2018.12.122

[198] Edemann-Callesen H Habelt B Wieske F Jackson M Khadka N Mattei D. Non-invasive modulation reduces repetitive behavior in a rat model through the sensorimotor cortico-striatal circuit. Transl Psychiatry. (2018) 8:11. 10.1038/s41398-017-0059-5 29317605 PMC5802458

[199] Behler N Leitner B Mezger E Weidinger E Musil R Blum B. Cathodal tDCS over motor cortex does not improve tourette syndrome: lessons learned from a case series. Front Behav Neurosci. (2018) 12:194. 10.3389/fnbeh.2018.00194 30197592 PMC6117531

